# Circulating myeloid-derived MMP8 in stress susceptibility and depression

**DOI:** 10.1038/s41586-023-07015-2

**Published:** 2024-02-07

**Authors:** Flurin Cathomas, Hsiao-Yun Lin, Kenny L. Chan, Long Li, Lyonna F. Parise, Johana Alvarez, Romain Durand-de Cuttoli, Antonio V. Aubry, Samer Muhareb, Fiona Desland, Yusuke Shimo, Aarthi Ramakrishnan, Molly Estill, Carmen Ferrer-Pérez, Eric M. Parise, C. Matthias Wilk, Manuella P. Kaster, Jun Wang, Allison Sowa, William G. Janssen, Sara Costi, Adeeb Rahman, Nicolas Fernandez, Matthew Campbell, Filip K. Swirski, Eric J. Nestler, Li Shen, Miriam Merad, James W. Murrough, Scott J. Russo

**Affiliations:** 1 Nash Family Department of Neuroscience, Icahn School of Medicine at Mount Sinai, New York, NY USA; 2 Brain and Body Research Center of the Friedman Brain Institute, Icahn School of Medicine at Mount Sinai, New York, NY USA; 3 Department of Oncological Sciences, Marc and Jennifer Lipschultz Precision Immunology Institute, Tisch Cancer Institute, Icahn School of Medicine at Mount Sinai, New York, NY USA; 4 Department of Biochemistry, Federal University of Santa Catarina, Santa Catarina, Brazil; 5 Microscopy CoRE and Advanced Bioimaging Center, Icahn School of Medicine at Mount Sinai, New York, NY USA; 6 Depression and Anxiety Center for Discovery and Treatment, Department of Psychiatry, Icahn School of Medicine of Mount Sinai, New York, NY USA; 7 Smurfit Institute of Genetics, Trinity College Dublin, Dublin, Ireland; 8 Cardiovascular Research Institute, Icahn School of Medicine at Mount Sinai, New York, NY USA

**Keywords:** Neuroimmunology, Depression

## Abstract

Psychosocial stress has profound effects on the body, including the immune system and the brain^1,2^. Although a large number of pre-clinical and clinical studies have linked peripheral immune system alterations to stress-related disorders such as major depressive disorder (MDD)^3^, the underlying mechanisms are not well understood. Here we show that expression of a circulating myeloid cell-specific proteinase, matrix metalloproteinase 8 (MMP8), is increased in the serum of humans with MDD as well as in stress-susceptible mice following chronic social defeat stress (CSDS). In mice, we show that this increase leads to alterations in extracellular space and neurophysiological changes in the nucleus accumbens (NAc), as well as altered social behaviour. Using a combination of mass cytometry and single-cell RNA sequencing, we performed high-dimensional phenotyping of immune cells in circulation and in the brain and demonstrate that peripheral monocytes are strongly affected by stress. In stress-susceptible mice, both circulating monocytes and monocytes that traffic to the brain showed increased *Mmp8* expression following chronic social defeat stress. We further demonstrate that circulating MMP8 directly infiltrates the NAc parenchyma and controls the ultrastructure of the extracellular space. Depleting MMP8 prevented stress-induced social avoidance behaviour and alterations in NAc neurophysiology and extracellular space. Collectively, these data establish a mechanism by which peripheral immune factors can affect central nervous system function and behaviour in the context of stress. Targeting specific peripheral immune cell-derived matrix metalloproteinases could constitute novel therapeutic targets for stress-related neuropsychiatric disorders.

## Abstract (web summary)

Serum MMP8 is increased in stress-susceptible mice following chronic stress and leads to brain structure and behavioural changes in mice.

## Main

Stress-related neuropsychiatric disorders such as MDD have a high worldwide prevalence and tremendous individual burden^4^. Although there are many effective treatments for MDD, more than a third of affected individuals do not achieve full remission following treatment with available antidepressant medications or established psychotherapeutic treatments^5^. One of the most important risk factors for depression is chronic psychosocial stress^6^. Therefore, elucidating the pathophysiological mechanisms underlying the effects of psychosocial stress is crucial to advancing our understanding of disorders such as MDD and ultimately developing treatment options and prevention strategies.

Immune interactions between the central nervous system (CNS) and peripheral organ systems are tightly regulated^7^. Psychosocial stress can affect this bidirectional communication profoundly, and disrupted neuroimmune interactions are increasingly recognized as important factors in the pathogenesis of stress disorders^8^. Chronic stress activates the innate immune system, resulting in mobilization of peripheral myeloid cells (for example, monocytes and neutrophils) and the production of pro-inflammatory cytokines, such as interleukin-6  (IL-6)^9,10^. In humans, it is well established that a subset of patients with stress-related neuropsychiatric disorders, such as MDD, display a state of chronic low-grade inflammation, characterized by increased circulating pro-inflammatory cytokines and leukocytosis^3^. In addition to these peripheral immune changes, stress disrupts the endothelial blood–brain barrier (BBB) in mice, allowing greater entry of circulating proteins directly into brain reward regions such as NAc^11,12^. Although these findings have provided important insights into the pathophysiology of stress and depression, we still know relatively little about the mechanisms by which these stress-induced immune changes affect neuronal function and ultimately behaviour.

In the brain, neurons and non-neuronal cells are separated by the extracellular space (ECS), which contains interstitial fluid, and the extracellular matrix (ECM), a dense scaffold of proteins and glycans secreted by neurons and glial cells^13^. ECM molecules have been shown to have an important role in homeostatic processes of the brain, including synaptic function^14^. ECM degradation and remodelling are regulated by various enzymes, such as matrix metalloproteinases (MMPs)^13,14^. MMPs in circulation have been associated with numerous inflammatory processes and disorders, such as cancer and myocardial infarction^15,16^. Although several studies have implicated CNS MMPs in synaptic remodelling and transmission by altering components of the ECM^17,18^, little is known about the effects of peripheral immune-derived MMPs in the context of psychosocial stress.

## Chronic stress affects monocytes

To investigate the effects of psychosocial stress on the immune system and how it affects the brain, we used the CSDS paradigm^19,20^. Interpersonal conflicts and social bullying are commonly experienced psychological stressors that can precipitate a major depressive episode^21,22^. The CSDS paradigm—one of the best-validated mouse models of psychosocial stress—consists of the experimental mice being subordinated by an aggressive CD-1 mouse through a combination of physical contact and sensory exposure over ten days. A majority of stressed mice develop a behavioural phenotype characterized by social avoidance^20^ and reduced preference for natural rewards (anhedonia), including impaired social reward^23^. They also develop several physiological disturbances, such as metabolic syndrome, systemic inflammation and gastrointestinal disturbances^10,24^. These mice are termed stress-susceptible (SUS) mice. However, a subset of mice shows behavioural and physiological profiles similar to unstressed control (CON) mice—these are termed resilient (RES) mice.

**Fig. 1:**
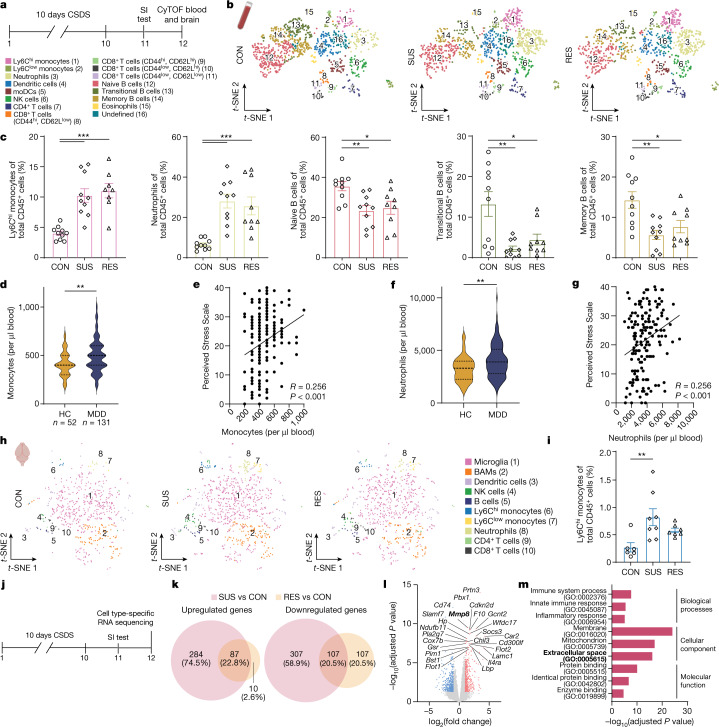
Stress increases monocyte numbers in the circulation and in the brain, and induces a pro-inflammatory transcriptional signature in monocytes of SUS mice. **a**, Experimental outline of CyTOF experiment. SI, social interaction. **b**,**h**, *t*-Distributed stochastic neighbour embedding (*t*-SNE) maps of CD45^+^ cells in blood (**b**) and brain (**h**). The colour of each cluster corresponds to the assigned cell type. BAMs, border-associated macrophages; moDCs, monocyte-derived dendritic cells; NK, natural killer. **c**, Frequencies of Ly6C^hi^ monocytes (*n* = 10 CON, 12 SUS and 10 RES), neutrophils (*n* = 10 CON, 10 SUS and 9 RES), naive B cells (*n* = 10 CON, 10 SUS and 9 RES), transitional B cells and memory B cells (*n* = 10 CON, 10 SUS and 9 RES) in circulation. One-way ANOVA with Bonferroni post hoc test. Each data point represents one biological sample; data are mean ± s.e.m. **d**,**f**, Number of monocytes (**d**) and neutrophils (**f**) in the circulation of patients with MDD compared with healthy controls (HC). *n* = 52 heathy controls and 131 MDD; two-tailed Student’s *t*-test. **e**,**g**, Correlation between perceived stress and monocyte numbers (**e**; *n* = 169 (HC and MDD)) and neutrophil numbers (**g**; *n* = 169 (HC and MDD)). Two-tailed Pearson correlation coefficient. **i**, Ly6C^hi^ monocytes in whole brain. *n* = 6 CON, 8 SUS and 7 RES. One-way ANOVA with Bonferroni post hoc test; each data point represents four pooled brains. Data are mean ± s.e.m. **j**, Experimental outline of cell type-specific RNA sequencing of Ly6C^hi^ and Ly6C^low^ monocytes, B cells and T cells from blood. **k**, Number of differentially expressed genes (adjusted *P* value < 0.05 and log_2_ fold change > |1|) in Ly6C^hi^ monocytes. **l**, The 25 most significantly differently expressed protein-coding genes in Ly6C^hi^ monocytes from SUS versus CON mice. **m**, Top three gene ontology (GO) terms from significantly upregulated genes in Ly6C^hi^ monocytes of SUS versus CON mice. **k**–**m**, *P* values adjusted for multiple comparisons. *P < 0.05, **P < 0.01, ***P < 0.001. Detailed statistics are in Supplementary Table 2. Source Data

**Extended Data Fig. 1:**
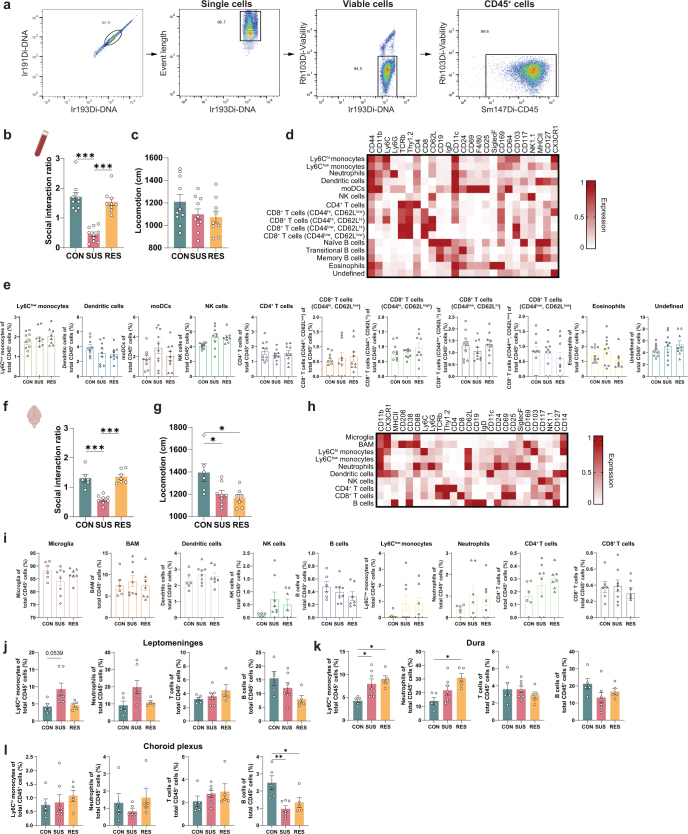
High-dimensional phenotyping of immune cells in blood, brain parenchyma, leptomeninges, dura and choroid plexus. **a**, Gating strategy for live CD45^+^ cells for the Cytometry by time of flight (CyTOF) experiment (**panels a**-**i**). **b**, Social interaction ratio (*n* = 10 CON, 10 SUS and 9 RES) and **c**, locomotion (*n* = 10 CON, 10 SUS and 9 RES) (behavioural data from blood CyTOF experiment). **d**, Marker expression and assigned leukocyte subpopulations in blood. **e**, Frequencies of Ly6C^low^ monocytes (*n* = 10 CON] 9 SUS and 8 [RES]), dendritic cells (*n* = 9 CON, 9 SUS and 9 [RES]), monocyte-derived dendritic cells (*n* = 9 CON, 10 SUS and 9 RES), natural killer cells (*n* = 9 CON, 9 SUS and 8 RES), CD4^+^ T cells (*n* = 10 CON, 9 SUS and 9 RES), CD8^+^ T cells (CD44^hi^, CD62L^low^) (*n* = 10 CON, 10 SUS and 9 RES), CD8^+^ T cells (CD44^hi^, CD62L^hi^) cells (*n* = 10 CON, 9 SUS and 9 RES), CD8^+^ T cells (CD44^low^, CD62L^hi^) (*n* = 10 CON, 10 SUS and 9 RES), CD8^+^ T cells (CD44^low^, CD62L^low^(*n* = 10 CON, 10 SUS and 9 RES), eosinophils (*n* = 10 CON, 10 SUS and 8 RES) and undefined (*n* = 9 CON, 10 SUS and 9 RES). **f**, Social interaction ratio (*n* = 6 CON, 8 SUS and 7 RES) and **g**, locomotion (*n* = 6 CON, 8 SUS and 7 RES) (behavioural data from brain CyTOF experiment). **h**, Marker expression and assigned leukocyte subpopulations of CD45^+^ brain leukocytes. **i**, Frequencies of microglia (*n* = 6 CON, 8 SUS and 7 RES), border-associated macrophages (*n* = 6 CON, 8 SUS and 7 RES), dendritic cells (*n* = 6 CON, 8 SUS and 7 RES), natural killer cells (*n* = 5 CON, 8 SUS and 7 RES), B cells (*n* = 6 CON, 8 SUS and 7 RES), Ly6C^low^ monocytes (*n* = 6 CON, 8 SUS, & 7 RES), neutrophils (*n* = 6 CON, 7 SUS, & 7 RES), CD4^+^ T cells (*n* = 6 CON, 8 SUS, & 7 RES), CD8^+^ T cells (*n* = 6 CON, 8 SUS and 7 RES) in whole brain tissue homogenates. Flow cytometry assessment of **j**, Ly6C^hi^ monocytes (*n* = 5 CON, 7 SUS and 5 RES), neutrophils (*n* = 5 CON, 7 SUS and 5 RES), T cells (*n* = 5 CON, 7 SUS and 5 RES), B cells (*n* = 5 CON, 7 SUS and 5 RES) in leptomeninges, **k**, Ly6C^hi^ monocytes (*n* = 5 CON, 7 SUS and 5 RES), neutrophils (*n* = 5 CON, 7 SUS and 5 RES), T cells (*n* = 5 CON, 7 SUS and 5 RES), B cells in dura (*n* = 5 CON, 7 SUS and 5 RES), **l**, Ly6C^hi^ monocytes (*n* = 5 CON, 7 SUS and 5 RES), neutrophils (*n* = 5 CON, 6 SUS and 5 RES), T cells (*n* = 5 CON, 7 SUS and 5 RES), B cells (*n* = 5 CON, 7 SUS and 5 RES) in choroid plexus. (One-way ANOVA with Bonferroni *post hoc* test **panels b**, **c**, **e**, **f**, **g**, **i**-**l**; for blood, each data point represents one biological sample; for brain, each data point represents four pooled brains; for leptomeninges, dura and choroid plexus, each data point represents three pooled samples). *P < 0.05, **P < 0.01, ***P < 0.001. Data are shown as mean ± s.e.m. Abbreviations: moDCs: Monocyte-derived dendritic cells; NK cells: Natural killer cells; BAM: Border-associated macrophages. Detailed statistics are in Supplementary Table 2. Source Data

We first performed high-dimensional phenotyping of immune cells of CON, SUS and RES mice from circulation and brain using mass cytometry (cytometry by time-of-flight (CyTOF)) (Fig. 1a and Extended Data Fig. 1a). On the basis of previous studies^25,26^, we compiled a panel of surface-receptor antibodies to capture the major immune cell lineages (Supplementary Table 1). In the blood, CSDS led to an increase in inflammatory Ly6C^hi^ monocytes and neutrophils, and a decrease in B cells in both SUS and RES mice (Fig. 1b,c and Extended Data Fig. 1b–e; detailed statistical information for each experiment is provided in Supplementary Table 2).

**Extended Data Fig. 2:**
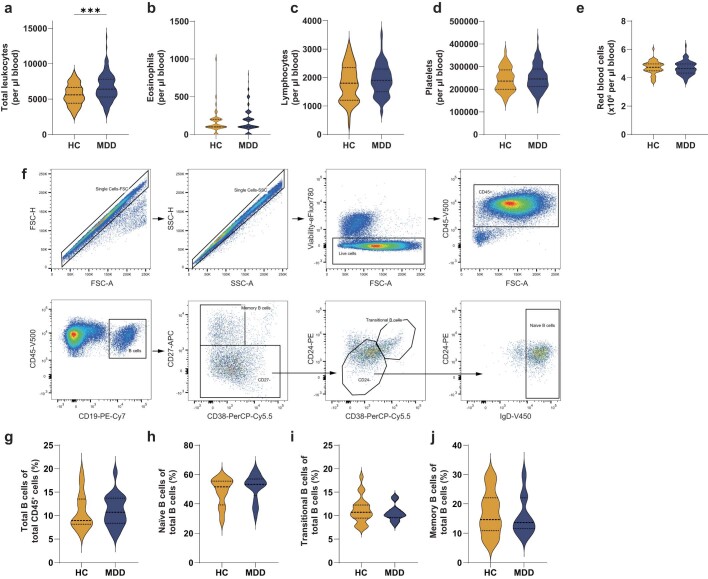
Leukocyte subpopulation frequencies in patients with major depressive disorder (MDD) compared to healthy controls (HC). Number of **a**, total leukocytes (*n* = 52 HC and 131 MDD), **b**, eosinophils (*n* = 52 and HC 131 MDD), **c**, lymphocytes (*n* = 52 HC and 131 MDD), **d**, platelets (*n* = 52 HC and 131 MDD), and **e**, red blood cells (*n* = 52 HC and 131 MDD), in patients with MDD compared to HC. **f**, Gating strategy of peripheral blood mononuclear cells to assess B cell subpopulations. **g**, Total B cells (*n* = 21 HC and 19 MDD), **h**, naïve B cells (*n* = 22 HC and 20 MDD), **i**, transitional B cells (*n* = 21 HC and 19 MDD) and **j**, memory B cells in MDD vs. HC (*n* = 21 HC and 19 MDD) (two-tailed Student’s t-test [panels **a**-**e**, **g**-**j**]). ***P < 0.001. Detailed statistics are in Supplementary Table 2. Source Data

To investigate whether these pre-clinical mouse findings also translate to human stress disorders, we assessed leukocyte subpopulations in blood from patients with MDD and healthy controls and found that patients with MDD displayed leukocytosis driven by increased numbers of monocytes and neutrophils but with no differences in lymphocytes, including total B cells, naive B cells, transitional B cells and memory B cells (Fig. 1d,f, Extended Data Fig. 2a–j and Supplementary Table 3). We also observed a significant positive correlation between the number of monocytes and neutrophils in circulation with perceived stress using the Perceived Stress Scale^27^, a clinically validated self-report measure of stress (Fig. 1e,g).

We assessed leukocytes in whole brains without meninges from mice following CSDS and observed a specific increase of pro-inflammatory Ly6C^hi^ monocytes in SUS mice, but not RES mice, compared with CON mice (Fig. 1h,i and Extended Data Fig. 1f–h). To prevent contamination with circulating leukocytes, brains were thoroughly perfused with PBS. Notably, we did not observe differences in other leukocytes or brain-resident immune cells, such as microglia or border-associated macrophages (Extended Data Fig. 1i). Finally, we assessed leukocyte subpopulations (monocytes, neutrophils, B cells and T cells) in leptomeninges, dura and choroid plexus after CSDS by flow cytometry. In the leptomeninges, we observed an increase in monocytes in SUS mice only, whereas in the dura there was an increase in both SUS and RES mice, compared with CON mice. No changes were observed in monocyte frequencies in the choroid plexus (Extended Data Fig. 1j–l).

**Extended Data Fig. 3:**
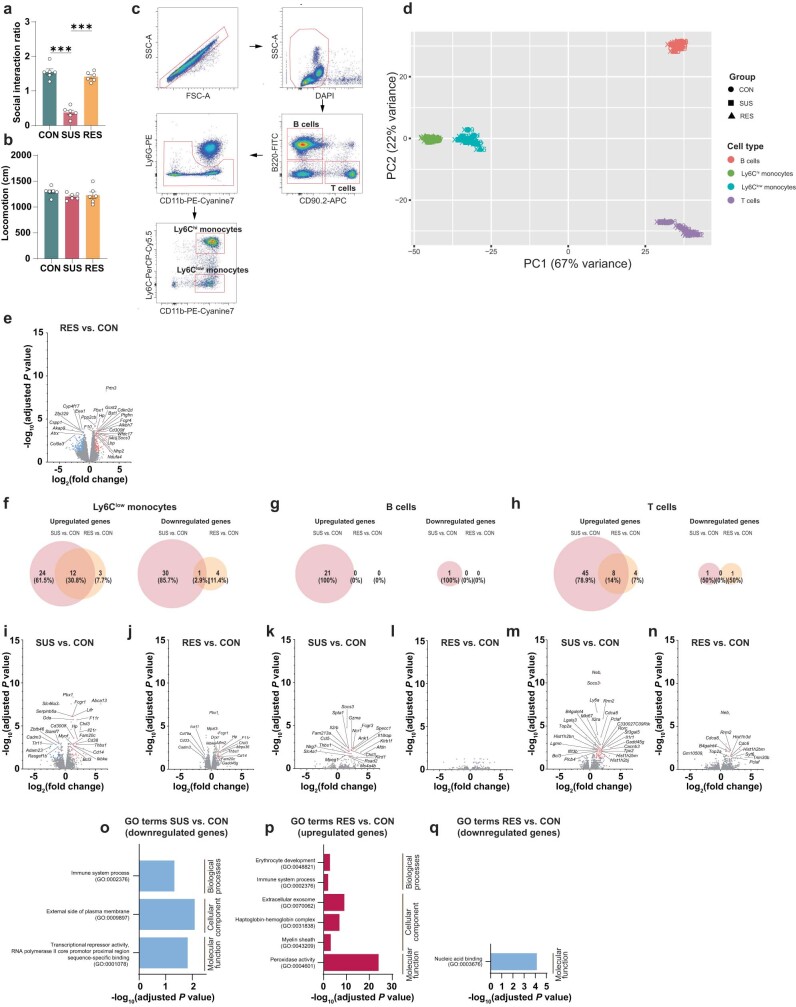
Cell type-specific RNA sequencing of leukocyte subpopulations in circulation. **a**, Social interaction ratio (*n* = 6 CON, 6 SUS and 6 [RES]) and **b**, locomotion (*n* = 6 CON, 6 SUS and 6 RES) (One-way ANOVA with Bonferroni *post hoc* test). **c**, Representative flow cytometry plots showing gating strategy for fluorescence-activated cell sorting of Ly6C^hi^ monocytes, Ly6C^low^ monocytes, B cells and T cells. **d**, Principal component analysis (PCA) plot performed on the top 2000 most variably expressed genes of sorted cells. **e**, Volcano plot showing the 25 most significantly differentially expressed protein coding genes in Ly6C^hi^ monocytes of RES vs. CON mice. Venn diagrams displaying number of differentially expressed genes (adjusted p-value < 0.05 and log_2_ fold change > |1 | ) in **f**, Ly6C^low^ monocytes, **g**, B cells and **h**, T cells. Volcano plots showing the 25 most significantly differentially expressed protein coding genes (if applicable) in Ly6C^low^ monocytes [**i**, SUS vs. CON, **j**, RES vs. CON], B cells [**k**, SUS vs. CON, **l**, RES vs. CON] and T cells [**m**, SUS vs. CON, **n**, RES vs. CON]. Top three gene ontology (GO) pathways (if applicable) in Ly6C^hi^ monocytes from **o**, significantly downregulated genes of SUS vs. CON mice, **p**, significantly upregulated genes in RES vs. CON and **q**, significantly downregulated genes in RES vs. CON mice. (all p-values adjusted for multiple comparisons in **panels e**-**q**) ***P < 0.001. Data are shown as mean ± s.e.m. Detailed statistics are in Supplementary Table 2. Source Data

To investigate differences in stress-induced transcriptional changes in the major circulating leukocyte subpopulations from CON, SUS and RES mice, we performed cell type-specific RNA sequencing of Ly6C^hi^ and Ly6C^low^ monocytes, B cells and T cells (Fig. 1j and Extended Data Fig. 3a–d). CSDS-induced changes in gene expression were most pronounced in Ly6C^hi^ monocytes, with a total of 785 differentially expressed genes in SUS versus CON mice and 311 differentially expressed genes in RES versus CON mice (adjusted *P* value < 0.05 and log_2_ fold change > |1|) (Fig. 1k,l and Extended Data Fig. 3e), with approximately 10 times fewer differentially expressed genes in the other cell types (Extended Data Fig. 3f–q). We then performed gene ontology (GO) enrichment analysis of biological processes, cellular components and molecular function. Genes upregulated in SUS versus CON mice were involved in GO biological processes such as innate immune response (GO:0045087) and inflammatory response (GO:0006954) and cellular components such as ECS (GO:0005615) (Fig. 1m). Together, CSDS increased monocyte numbers in circulation and in the brain and induced a pro-inflammatory transcriptional signature in SUS mice. These findings, which emphasize the role of peripheral myeloid cells in stress-linked disorders such as MDD, are in line with several pre-clinical studies^9,10,28^ and studies in humans^29,30^. Therefore, we focused on elucidating the mechanisms by which peripheral monocytes can affect neuronal function and behaviour.

## Brain-trafficking monocytes express *Mmp8*

**Fig. 2:**
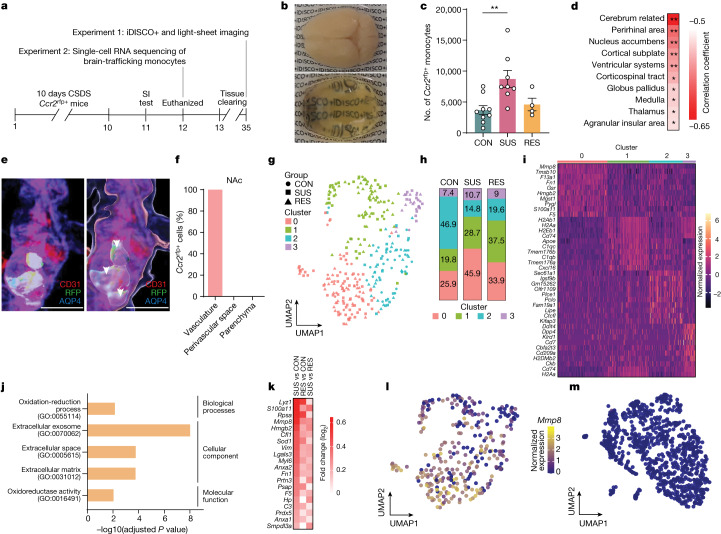
Increased expression of matrix metalloproteinase 8 mRNA (*Mmp8*) in brain-trafficking Ly6C^hi^ monocytes of SUS mice. **a**, Outline of iDISCO+ brain clearing and light-sheet imaging, and single-cell RNA-sequencing experiments. **b**, Representative images of a mouse brain before (top) and after (bottom) iDISCO+ clearing. **c**, Number of *Ccr2*^*rfp*+^ monocytes in whole brain of CON, SUS and RES mice (*n* = 9 CON, 8 SUS and 4 RES mice). One-way ANOVA with Bonferroni post hoc test. Data are mean ± s.e.m. **d**, Top ten most significant correlations between brain-trafficking monocytes and SI ratio. Lower correlation coefficients (red) indicate that higher numbers of monocytes are associated with greater social avoidance. Two-tailed Pearson correlation coefficient. **e**, Representative *z*-stack (3–4 slices per brain in three mice) (left) and corresponding three-dimensional reconstruction (right) of a confocal image of the NAc. Brain slices were stained with antibodies to markers for blood vessels (CD31, red), *Ccr2*^*rfp*+^ monocytes (RFP, green and arrowheads) and astrocytes (AQP4, blue). Scale bars, 10 µm. **f**, Percentage of *Ccr2*^*rfp*+^ monocytes located in the brain vasculature, the perivascular space and parenchyma. **g**, Uniform manifold approximation and projection (UMAP) representation of four clusters identified using Seurat clustering. **h**, Relative abundance (as a percentage) of the number of cells per cluster in CON, SUS and RES mice. **i**, Heat map of top ten cluster-defining protein-coding genes. **j**, GO terms of significantly (adjusted *P* value < 0.05) upregulated genes of cluster 0. **k**, Expression of genes within the GO terms ‘extracellular space’ and ‘extracellular matrix’ compared between SUS versus CON, RES versus CON and SUS versus RES mice. **l**,**m**, Feature plots of normalized gene expression of *Mmp8* in *Ccr2*^*rfp*+^ monocytes in the brain (**l**) and brain-resident immune cells in the NAc (**m**). **P < 0.01. Detailed statistics are in Supplementary Table 2. Source Data

**Extended Data Fig. 4:**
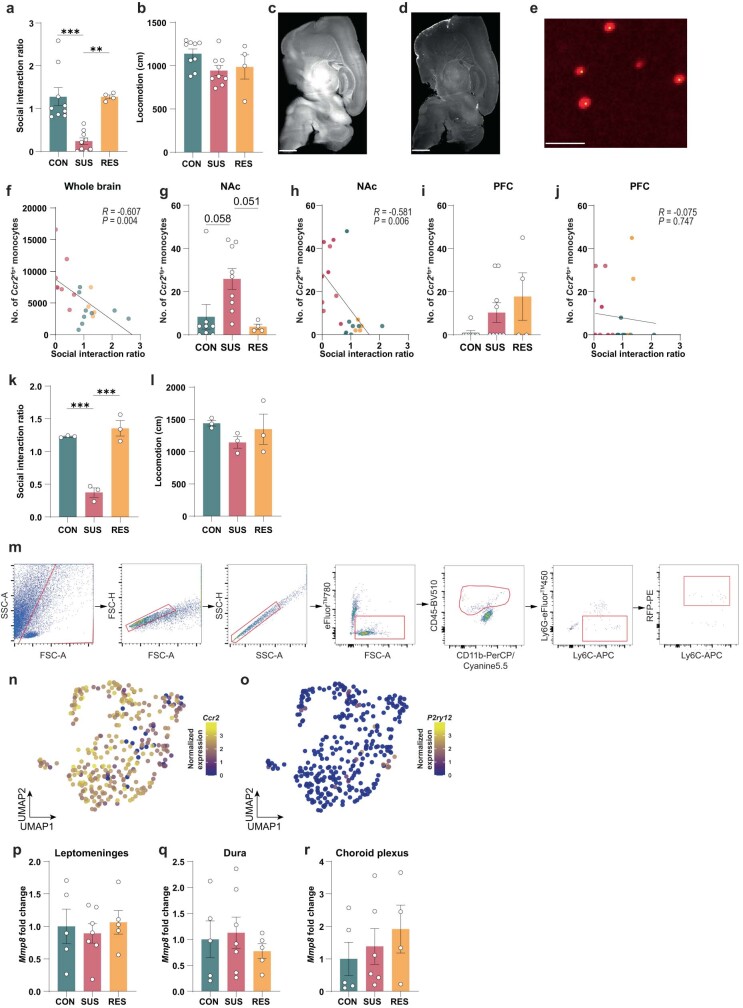
Anatomical mapping and transcriptional analyses of brain-trafficking *Ccr2*^rfp+^ monocytes. **a**, Social interaction ratio (*n* = 9 CON, 9 SUS and 4 RES) and **b**, locomotion (*n* = 9 CON, 9 SUS and 4 RES) (One-way ANOVA with Bonferroni *post hoc* test). Representative images (from 22 total brains) from light-sheet imaging of **c**, auto-fluorescent signal (scale bar: 1000 µm) and **d**, monocyte signal (scale bar: 1000 µm). **e**, brain-trafficking monocytes (red) in the nucleus accumbens (NAc) showing accurate cell detection (yellow) by the automated software ClearMap (scale bar: 30 µm). Number of brain-trafficking *Ccr2*^rfp+^ monocytes in the **g**, NAc (*n* = 8 CON, 9 SUS and 4 RES) and **i**, prefrontal cortex (PFC) (*n* = 8 CON, 9 SUS and 4 RES) (One-way ANOVA with Bonferroni *post hoc* test) and correlations with social interaction ratio in **f**, whole brain (*n* = 21 CON, SUS and RES), **h**, NAc (*n* = 21 CON, SUS and RES) and **j**, PFC (*n* = 21 CON, SUS and RES) (Two-tailed Pearson correlation coefficient, two-tailed, **panels f**-**j**). **I k**, Social interaction ratio (*n* = 3 CON, 3 SUS and 3 RES) and **l**, locomotion (*n* = 3 CON, 3 SUS and 3 RES) (One-way ANOVA with Bonferroni *post hoc* test). **m**, Gating strategy for fluorescence-activated cell sorting of brain-trafficking monocytes. Feature plots of the **n**, monocyte-enriched gene *Ccr2* and **o**, the microglia-enriched gene *P2ry12*. *Mmp8* gene expression levels in **p**, leptomeninges (*n* = 5 CON, 7 SUS and 5 RES), **q**, dura (*n* = 5 CON, 7 SUS and 5 RES) and **r**, choroid plexus (*n* = 5 CON, 6 SUS and 4 RES) (One-way ANOVA with Bonferroni *post hoc* test; for **p**-**r**, three samples were pooled). **P < 0.01, ***P < 0.001. Data in **panels a**, **b**, **g**, **i**, **j**, **k**, **l** and **p**-**r** are shown as mean ± s.e.m. Statistics are shown in Supplementary Table 2. Source Data

First, we sought to investigate the exact locations where inflammatory Ly6C^hi^ monocytes traffic in the brain (hereafter referred to as brain-trafficking monocytes). We performed detailed anatomic mapping of monocytes with the whole-brain tissue-clearing method iDISCO+^31^. Using the *Ccr2*^*rfp*^ reporter line, in which monocytes express a red fluorescent protein, we cleared brains from CON, SUS and RES mice, then performed light-sheet microscopy and registered the samples to the Allen Brain Atlas using ClearMap^31^ (Fig. 2a,b and Extended Data Fig. 4a–e). We first analysed total cells in whole brains and confirmed our CyTOF data showing increased monocytes only in SUS mice and a negative correlation with social interaction (SI) ratio (Figs. 1i and 2c and Extended Data Fig. 4f). Next, we examined the correlation between monocytes in specific brain regions with social avoidance behaviour. Cell counts in limbic brain areas such as the NAc were highly correlated with SI ratio, with higher numbers of monocytes in the NAc correlating with greater social avoidance behaviour (Fig. 2d, Extended Data Fig. 4g,h and Supplementary Table 4). The NAc is a stress-responsive brain region that is central to processing rewarding and aversive stimuli^32^, and is critical in mediating depression symptomatology^33^. In contrast to the NAc, we did not observe increased monocyte trafficking to the prefrontal cortex (PFC) or a significant correlation between SI ratio and monocytes in the PFC (Extended Data Fig. 4i,j). Confocal microscopy revealed that monocytes were attached to the vasculature in NAc but did not infiltrate the brain parenchyma or perivascular space (Fig. 2e,f).

**Extended Data Fig. 5:**
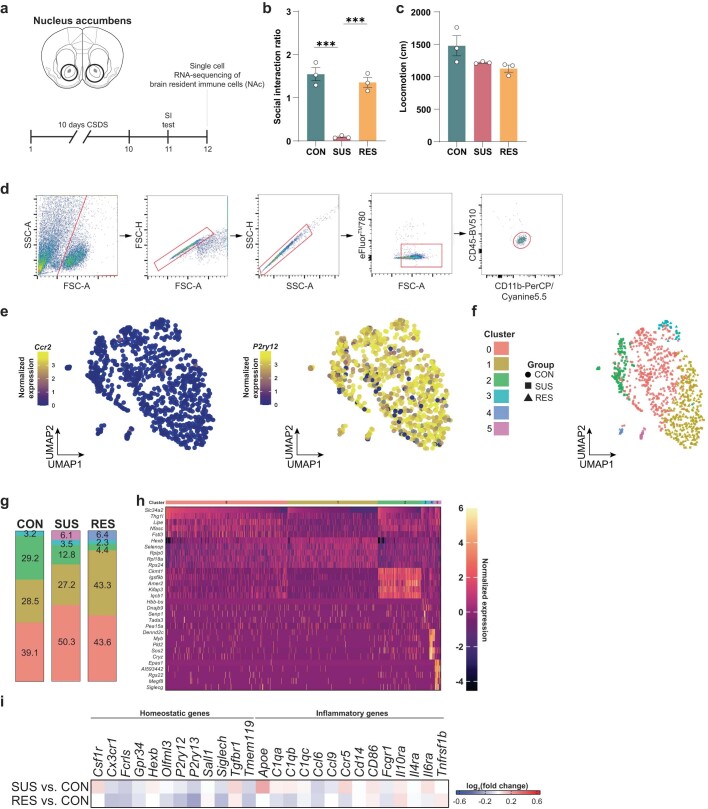
Single-cell RNA sequencing of brain-resident myeloid cells in the nucleus accumbens (NAc). **a**, Experimental outline. **b**, Social interaction ratio (*n* = 3 CON, 3 SUS and 3 RES) and **c**, locomotion (*n* = 3 CON, 3 SUS and 3 RES) (One-way ANOVA with Bonferroni *post hoc* test). **d**, Gating strategy for fluorescence-activated cell sorting of brain-resident myeloid cells. **e**, Feature plots (Uniform manifold approximation and projection [UMAP]). **f**, UMAP representation of 6 clusters identified using Seurat clustering. **g**, Relative (%) abundance of number of cells per cluster in CON, SUS and RES mice. Cluster 5 was specific to SUS mice and cluster 4 was specific to RES mice, however, gene ontology analyses did not reveal any significantly enriched pathways. **h**, Heat map of top five cluster defining protein coding genes. **i**, heatmap of homeostatic and inflammatory genes. There were no significant differences (*P* value adjusted <0.05) in these homeostatic or inflammatory genes. ***P < 0.001. Data are shown as mean ± s.e.m. Detailed statistics are in Supplementary Table 2. Source Data

Next, we investigated how brain-trafficking monocytes contribute to stress-induced social avoidance and performed single-cell RNA sequencing of brain-trafficking monocytes after CSDS (Fig. 2a and Extended Data Fig. 4k–o). Unsupervised clustering of *Ccr2*^*rfp*+^ monocytes revealed four unique clusters based on their transcriptional profiles (Fig. 2g). Cluster 0 was enriched in SUS mice relative to CON and RES mice (Fig. 2h). To determine cluster-defining genes, we performed a differential gene expression analysis by investigating differentially expressed genes between clusters and total genes (adjusted *P* value < 0.05) (Fig. 2i). Several genes known to be involved in inflammatory processes were found to be upregulated in Cluster 0: for example, genes encoding S100 proteins, including S100A6 and S100A11^34,35^, lysozyme (*Lyz1*), an antimicrobial protein critical for host defence^36^, and annexins (*Anxa1* and *Anxa2*). GO term analysis of upregulated genes of cluster 0 revealed involvement in oxidation–reduction process (GO:0055114), ECS (GO:0005615) and ECM (GO:0031012) (Fig. 2j). One of the top genes enriched in these ECS and ECM pathways was *Mmp8*, which encodes matrix metalloproteinase 8 (Fig. 2k,l). Of note, *Mmp8* was also one of the top differentially expressed genes in circulating Ly6C^hi^ monocytes from SUS versus CON mice, as was the GO term extracellular space (Fig. 1l,m). We did not observe increased *Mmp8* gene expression in monocytes from leptomeninges, dura or choroid plexus in SUS mice, indicating that the source of MMP8 in the brain is probably from circulating monocytes (Extended Data Fig. 4p–r). We also performed single-cell RNA sequencing of brain-resident immune cells in the NAc (Extended Data Fig. 5a–e), however, we did not observe any stress-induced changes in homeostatic or inflammatory gene signatures (Extended Data Fig. 5f–i). Whereas previous studies in mice reported stress-induced morphological, transcriptional and functional changes indicative of an inflammatory signature in microglia from other brain regions^37,38^, to our knowledge, this is the first study investigating cell type-specific gene expression signatures at the single-cell level in NAc microglia. This finding suggests that there are brain region-specific differences in microglial reactivity to stress, in line with recent studies showing substantial heterogeneity in microglia across brain regions^39,40^. Our results are also consistent with recent single-cell studies conducted in postmortem brain samples from people with MDD, where no evidence of pro-inflammatory microglia signatures was found^40,41^.

MMP8 belongs to the group of collagenases and is derived and secreted largely from neutrophils and monocytes^42^. Our data and previous studies suggest that unlike many other MMPs, MMP8 is not produced or secreted by any cells of the CNS, including brain-resident myeloid cells^42^ (Fig. 2m). Of note, in a whole-blood gene expression study, *MMP8* was among the top upregulated genes in patients with MDD compared with healthy controls^43^. In addition, a single-nucleotide polymorphism in the coding region of *Mmp8* has been associated with MDD^44^. However, the underlying mechanisms linking MMP8 with MDD have not been explored. Activated MMP8 can cleave a wide range of ECM components, such as collagens, fibronectins, tenascins and aggrecan, many of which are components of the brain ECM^14,42^.

## MMP8 correlates with brain ECS changes

**Fig. 3:**
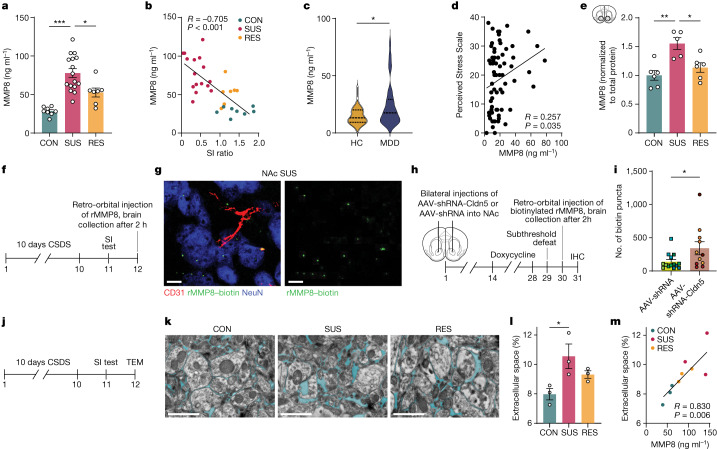
Stress-induced increase in MMP8 is associated with altered ECS in the NAc. **a**, Plasma levels of MMP8 after CSDS. One-way ANOVA with Bonferroni post hoc test (*n* = 8 CON, 16 SUS and 7 RES mice). **b**, Correlation of MMP8 in circulation with SI ratio (*n* = 31 CON, SUS and RES mice; two-tailed Pearson correlation coefficient). **c**, Serum MMP8 levels in patients with MDD (*n* = 40) and healthy controls (HC) (*n* = 29). Two-tailed Student’s *t*-test. **d**, Correlation between MMP8 and Perceived Stress Scale (*n* = 68 HC and MDD; two-tailed Pearson correlation coefficient). **e**, Normalized MMP8 protein levels in NAc lysates of CON (*n* = 6), SUS (*n* = 5) and RES (*n* = 6) mice. One-way ANOVA with Bonferroni post hoc test; each data point represents three pooled NAcs. **f**,**g**, Experimental outline (**f**) and representative immunofluorescence image of the NAc of SUS mice that were injected retro-orbitally with biotinylated rMMP8 (**g**). Scale bars, 10 µm. Three to four slices per brain in three mice. **h**,**i**, Experimental outline (**h**) and number of biotin puncta in the NAc in AAV-shRNA versus AAV-shRNA-Cldn5-injected mice (**i**). Each data point represents one slice; slices from the same mice have the same colour (AAV-shRNA: 5 mice, 14 brain slices; AAV-shRNA-Cldn5: 4 mice, 11 brain slices). Linear mixed model. IHC, immunohistochemistry. **j**,**k**, Outline of transmission electron microscopy experiment to assess the ECS in the NAc (**j**) and representative transmission electron microscopy (TEM) images from CON, SUS and RES mice (**k**). Scale bars, 1 µm. **l**, Quantification of the ECS relative to total brain area (*n* = 3 CON, 3 SUS and 3 RES mice). One-way ANOVA with Bonferroni post hoc test. **m**, Correlation between plasma MMP8 levels and ECS volume fraction (*n* = 9 CON, SUS and RES mice). Two-tailed Pearson correlation coefficient. Data are mean ± s.e.m. *P < 0.05, **P < 0.01, ***P < 0.001. Detailed statistics are in Supplementary Table 2. Source Data

**Extended Data Fig. 6:**
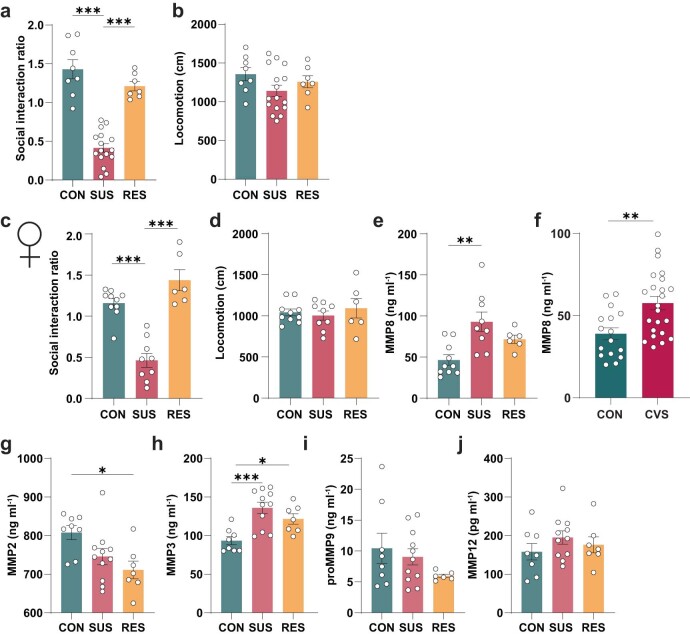
Stress increases matrix metalloproteinase 8 (MMP8) in both male and female mice. **a**, Social interaction ratio (*n* = 8 CON, 16 SUS and 7 RES) and **b**, locomotion (*n* = 8 CON, 16 SUS and 7 RES) (One-way ANOVA with Bonferroni *post hoc* test). Defeat characteristics of female mice after 10 days of chronic social defeat stress (CSDS): **c**, social interaction ratio (*n* = 10 CON, 9 SUS and 6 RES), **d**, locomotion (*n* = 10 CON, 9 SUS and 6 RES) (One-way ANOVA with Bonferroni *post hoc* test). **e**, Plasma levels of MMP8 in SUS compared to CON female mice after 10 days of CSDS (*n* = 10 CON, 9 SUS and 6 RES) (One-way ANOVA with Bonferroni *post hoc* test). **f**, MMP8 plasma levels of female mice that underwent a 21-day paradigm of chronic variable stress (CVS) compared to non-stressed control mice (*n* = 16 CON and 23 CVS) (two-tailed Student’s t-test). Plasma levels of **g**, MMP2 (*n* = 8 CON, 11 SUS and 7 [RES]), **h**, MMP3 (*n* = 8 CON, 11 SUS and 7 RES), **i**, proMMP9 (*n* = 8 CON, 11SUS and 6 RES) and **j**, MMP12 (*n* = 8 CON, 11 SUS and 7 RES) in CON, SUS and RES male mice after 10 days of CSDS (One-way ANOVA with Bonferroni *post hoc* test). *P < 0.01, **P < 0.01, ***P < 0.001. Data are shown as mean ± s.e.m. Detailed statistics are in Supplementary Table 2. Source Data

**Extended Data Fig. 7:**
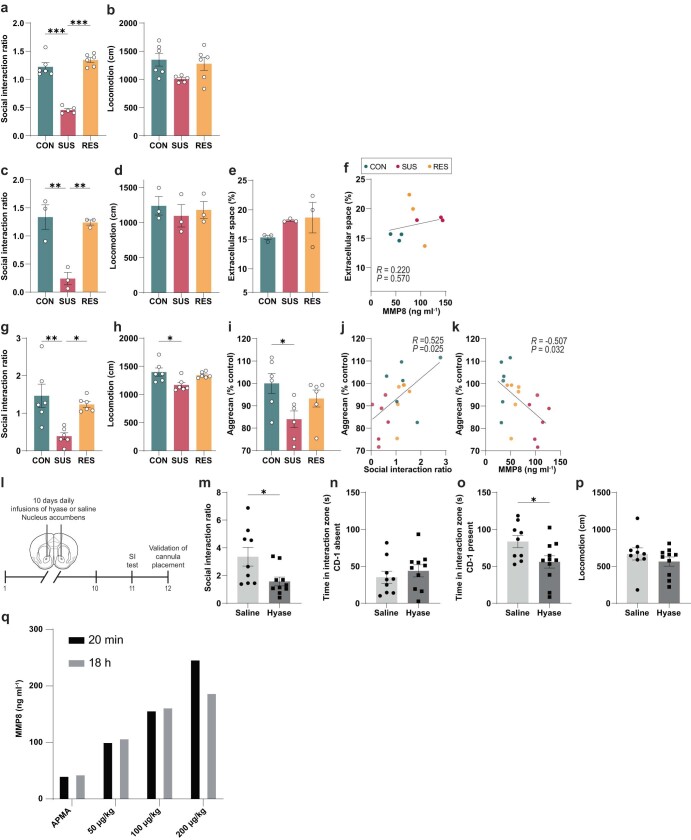
Chronic social defeat stress and brain extracellular space. **a**, Social interaction ratio (*n* = 6 CON, 5 SUS and 6 RES) and **b**, locomotion (*n* = 6 CON, 5 SUS and 6 RES) from mice from which levels of MMP8 were analysed in the NAc. **c**, Social interaction ratio (*n* = 3 CON, 3 SUS and 3 RES) and **d**, locomotion (*n* = 3 CON, 3 SUS and 3 RES) of mice analysed for the transmission electron microscopy experiment. **e**, Quantification of the extracellular space relative to total brain in prefrontal cortex (PFC) (*n* = 3 CON, 3 SUS and 3 RES). **f**, Correlation between plasma MMP8 levels and extracellular space volume fraction in PFC. **g**, Social interaction ratio (*n* = 6CON, 6 SUS and 6 RES) and **h**, locomotion of mice analysed for the aggrecan western blots (*n* = 6 CON 6 SUS and 6 RES). **i**, Aggrecan levels in the NAc after chronic social defeat stress (*n* = 6 CON, 6 SUS and 6 RES), correlations between **j**, aggrecan levels and social interaction ratio and **k**, aggrecan levels and circulating MMP8. **l**, Experimental design of hyaluronidase (hyase) infusion experiment. **m**, Social interaction ratio (*n* = 9, Saline and 10 Hyase), **n**, time spent in the interaction zone when the target mouse is absent (*n* = 9, Saline and 10 Hyase), **o**, time spent in the interaction zone when the target mouse is present (*n* = 9, Saline and 11 Hyase), **p**, locomotion after 10 days of chronic hyase injection (*n* = 9, Saline, 10 Hyase). **q**, Different doses of rMMP8 or vehicle (4-aminophenylmercuric acetate, APMA) were injected i.p. and blood was collected 20 min or 18 h after the injection followed by MMP8 measurement in plasma. (One-way ANOVA with Bonferroni *post hoc* test [**panels a**-**e**, **g**-**i**], Two-tailed Pearson correlation coefficient, two-tailed, [**panels f**, **j**, **k**], two-sided Student’s t-test [**panels m**-**p**]). *P < 0.05, **P < 0.01, ***P < 0.001. Data are shown as mean ± s.e.m. Detailed statistics are in Supplementary Table 2. Source Data

To test whether circulating myeloid-derived MMP8 can promote stress susceptibility, we first confirmed the stress-induced increase in MMP8 at the protein level in plasma after CSDS (Fig. 3a and Extended Data Fig. 6a,b), and showed that MMP8 levels were negatively correlated with SI ratio (Fig. 3b). We further demonstrated that both 10 days of CSDS and 21 days of chronic variable stress increased plasma levels of MMP8 in female mice (Extended Data Fig. 6c–f). Next, we measured plasma levels of other MMP proteins such as MMP2, MMP3, proMMP9 and MMP12 in the same mice as shown in Fig. 3a after 10 days of CSDS. Although we did observe a modest increase of MMP3, we observed similar changes in both SUS and RES mice compared to CON mice, confirming that only MMP8 is uniquely upregulated in SUS mice but not in CON or RES mice (Extended Data Fig. 6g–j). Finally, we validated the increased MMP8 in serum from patients with MDD compared with healthy controls (Fig. 3c and Supplementary Table 5) and found a positive correlation with self-reported perceived stress (Fig. 3d). We then confirmed that MMP8 was increased in the NAc but not the PFC of SUS mice following CSDS (Fig. 3e and Extended Data Fig. 7a,b). By retro-orbitally injecting biotinylated mouse recombinant MMP8 (rMMP8) into stress-susceptible mice (Fig. 3f), we showed that peripheral MMP8 can access the brain parenchyma (Fig. 3g). How MMP8 accesses the brain parenchyma is an important question. Our lab has recently shown that CSDS leads to reduced expression of the endothelial tight junction protein claudin 5 (CLDN5) and increased permeability of the BBB in SUS mice compared with RES and CON mice^11^. We therefore hypothesized that MMP8 may access the brain through a damaged BBB. To demonstrate this, we experimentally disrupted the BBB by depleting *Cldn5* in the NAc via stereotaxic injection of an adeno-associated virus expressing either a *Cldn5*-targeting short hairpin RNA (shRNA) transcript (AAV-shRNA-Cldn5) or a control nontargeting transcript (AAV-shRNA). After a subthreshold social defeat, we injected biotinylated rMMP8 retro-orbitally into circulation, thoroughly perfused the mouse brain with PBS to remove circulating rMMP8, and performed immunohistochemical staining and quantification of rMMP8 (Fig. 3h). We found that knockdown of *Cldn5* increased levels of biotinylated rMMP8 in the brain parenchyma compared with mice injected with the nontargeting vector (Fig. 3i).

We then investigated whether CSDS affects the ECS of the brain. First, we assessed the percentage of ECS using transmission electron microscopy imaging of NAc and PFC tissue sections from mice following CSDS (Fig. 3j). Compared with unstressed controls, SUS mice showed increased ECS volume fractions in the NAc, whereas we did not observe any group differences in the PFC (Fig. 3k,l and Extended Data Fig. 7c–e). Increases in brain ECS volume have been described in other CNS pathologies, such as neurodegenerative disorders, and can be a result of disrupted cell attachments associated with degradation of the ECM^45^. Notably, ECS volume fractions in the NAc, but not PFC, positively correlated with peripheral MMP8 in the same mice (Fig. 3m and Extended Data Fig. 7f). MMP8 has been shown to proteolytically degrade aggrecan—an important protein of the ECS—which binds other glycoproteins and proteoglycans such as hyaluronic acid to the cell surface of neurons and glial cells and is therefore crucial in organizing the neuronal ECS^46,47^. Studies in a mouse model of chronic pain revealed that in the hippocampus, increased MMP8 was associated with a decrease in aggrecan and structural changes in the ECM^48^. We therefore quantified the amount of aggrecan in the NAc of CON, SUS and RES mice and found a downregulation in SUS mice compared with CON mice (Extended Data Fig. 7g–i), a positive correlation between aggrecan levels and SI ratio (Extended Data Fig. 7j), and a negative correlation between peripheral MMP8 and aggrecan (Extended Data Fig. 7k). Finally, to test whether directly altering the ECS affects social behaviour, we implanted bilateral cannulae into the NAc and, to mimic the chronic nature of the CSDS, infused hyaluronidase (a hyaluronic acid-degrading enzyme) or vehicle once daily for ten days (Extended Data Fig. 7l). Hyaluronidase can break down hyaluronic acid in the ECM, resulting in increased brain ECS volume and has been shown to alter neurophysiology in rodents^49–51^. We found increased social avoidance behaviour in hyaluronidase-treated mice compared with vehicle-injected mice (Extended Data Fig. 7m–p), further supporting our data suggesting that changes in the NAc ECS are linked to changes in social behaviour.

## MMP8 regulates social avoidance

**Fig. 4:**
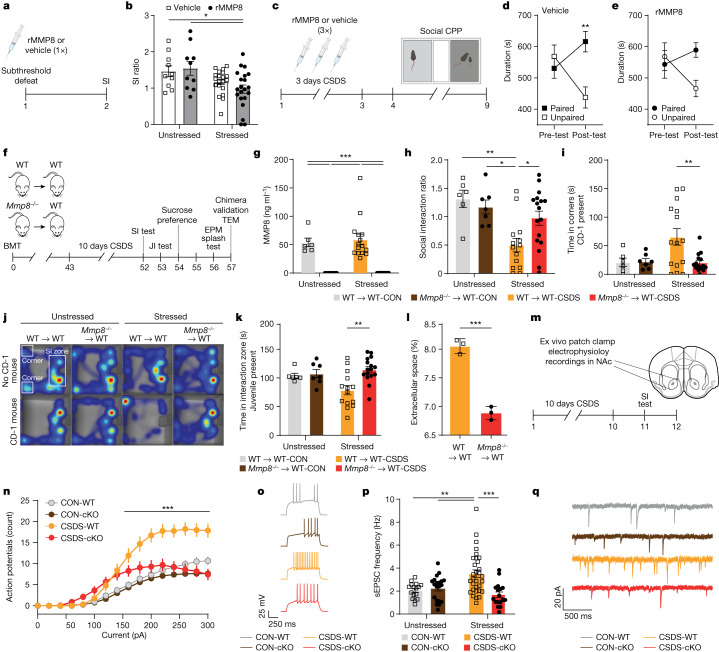
Circulating MMP8 is causally linked to stress-induced social avoidance behaviour, alterations in brain ECS and neurophysiology. **a**, Experimental outline. **b**, SI ratio of mice injected with rMMP8 followed by a subthreshold defeat. *n* = 10 unstressed vehicle, 10 unstressed rMMP8, 20 stressed vehicle and 21 stressed rMMP8. **c**, Experimental outline. **d**,**e**, sCPP of vehicle-injected mice (**d**; *n* = 18) and rMMP8-injected mice (**e**; *n* = 16). **f**, Experimental outline. EPM, elevated plus maze test; JI, juvenile interaction; WT, wild type. **g**, Plasma levels of MMP8 (*n* = 6 WT → WT-CON, 7 *Mmp8*^−/−^ → WT-CON, 15 WT → WT-CSDS and 17 *Mmp8*^−/−^ → WT-CSDS). **h**,**i**, SI ratio (**h**; *n* = 6 WT → WT-CON, 7 *Mmp8*^−/−^ → WT-CON, 14 WT → WT-CSDS and 17 *Mmp8*^−/−^ → WT-CSDS) and time spent in the corner (**i**; *n* = 6 WT → WT-CON, 7 *Mmp8*^−/−^ → WT-CON, 15 WT → WT-CSDS and 15 *Mmp8*^−/−^ → WT-CSDS). **j**, Representative heat maps of behaviour during SI test. **k**, Time spent in the interaction zone when the novel juvenile mouse was present (*n* = 6 WT → WT-CON, 7 *Mmp8*^−/−^ → WT-CON, 14 WT → WT-CSDS and 16 *Mmp8*^−/−^ → WT-CSDS). **l**, Quantification of the ECS in the NAc relative to total brain area in WT mice compared with *Mmp8*^−/−^ chimeras (*n* = 3 per group). **m**, Experimental outline. **n**, Action potentials in constitutive *Mmp8*^−/−^ knockout (cKO) and wild-type mice after 10 days of CSDS (*n* = 6 CON-WT, 5 CON-cKO, 5 CSDS-WT and 5 CSDS-cKO; 3 slices per mouse). **o**, Representative traces of action potentials evoked upon a +200 pA step of depolarizing current. **p**, Frequencies of spontaneous excitatory postsynaptic currents (sEPSCs) in cKO *Mmp8*^−/−^ and WT mice after 10 days of CSDS (*n* = 6 CON-WT, 5 CON-cKO, 5 CSDS-WT and 5 CSDS-cKO; 3 slices per mouse). **q**, Representative traces of sEPSCs. Two-way ANOVAs followed by Tukey’s post hoc test (**b**,**d**,**g**–**i**,**k**,**n**,**p**) or two-sided Student’s *t*-test (**l**). Data are mean ± s.e.m. *P < 0.05, **P < 0.01, ***P < 0.001. Detailed statistics are in Supplementary Table 2. Source Data

Next, we determined whether MMP8 is causally linked to stress-induced social avoidance. First, we tested whether the combination of an intraperitoneal injection of rMMP8 (at a dose that leads to similar plasma levels of MMP8 as observed in SUS mice after CSDS (Extended Data Fig. 7q)) coupled with subthreshold social defeat promotes stress susceptibility (Fig. 4a). We found that the combination of rMMP8 and subthreshold stress led to a lower SI ratio compared with unstressed mice (Fig. 4b). We then tested whether rMMP8 also changed social preference by testing mice with a non-threatening same-sex juvenile mouse using a social conditioned place preference (sCPP) test (Fig. 4c). This paradigm has been used historically to assess social reward^52^. Whereas mice that received vehicle injections during three days of a subthreshold CSDS formed a preference for the chamber that was previously paired with the juvenile mouse (Fig. 4d), the social preference was attenuated in mice that received rMMP8 (Fig. 4e).

**Extended Data Fig. 8:**
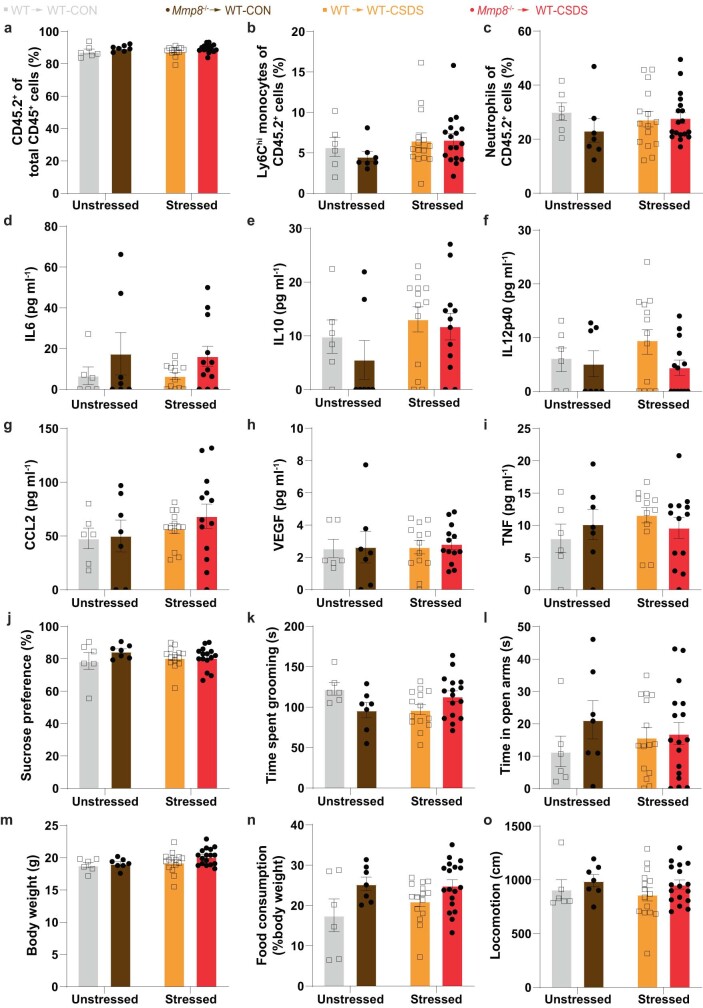
Bone marrow chimera experiments. **a**, Chimerism (CD45.2^+^ vs. total CD45^+^ cells) of unstressed and stressed WT and *Mmp8*^−/−^ transplants (*n* = 6 WT → WT-CON, 7 *Mmp8*^−/−^ → WT-CON, 15 WT → WT-CSDS and 17 *Mmp8*^−/−^ → WT-CSDS). Frequencies of circulating **b**, Ly6C^hi^ monocytes (*n* = 6 WT → WT-CON, 7 *Mmp8*^−/−^ → WT-CON, 15 WT → WT-CSDS and 17 *Mmp8*^−/−^ → WT-CSDS) and **c**, neutrophils (*n* = 6 WT → WT-CON, 7 *Mmp8*^−/−^ → WT-CON, 15 WT → WT-CSDS and 17 *Mmp8*^−/−^ → WT-CSDS). Plasma levels of **d**, Interleukin 6 (IL6) (*n* = 6 WT → WT-CON, 7 *Mmp8*^−/−^ → WT-CON, 12 WT → WT-CSDS and 12 *Mmp8*^−/−^ → WT-CSDS), **e**, IL10 (*n* = 6 WT → WT-CON, 7 *Mmp8*^−/−^ → WT-CON, 13 WT → WT-CSDS and 12 *Mmp8*^−/−^ → WT-CSDS), **f**, IL12p40 (*n* = 6 WT → WT-CON, 7 *Mmp8*^−/−^ → WT-CON, 13 WT → WT-CSDS and 13 *Mmp8*^−/−^ → WT-CSDS), **g**, C-C motif chemokine ligand 2 (CCL2) (*n* = 6 WT → WT-CON, 7 *Mmp8*^−/−^ → WT-CON, 13 WT → WT-CSDS and 13 *Mmp8*^−/−^ → WT-CSDS), **h**, Vascular endothelial growth factor (VEGF) (*n* = 6 WT → WT-CON, 7 *Mmp8*^−/−^ → WT-CON, 13 WT → WT-CSDS and 13 *Mmp8*^−/−^ → WT-CSDS) and **i**, Tumor necrosis factor (TNF) (*n* = 6 WT → WT-CON, 7 *Mmp8*^−/−^ → WT-CON, 12 WT → WT-CSDS and 13 *Mmp8*^−/−^ → WT-CSDS). **j**, Sucrose preference test (*n* = 6 WT → WT-CON, 7 *Mmp8*^−/−^ →WT-CON, 14 WT → WT-CSDS and 15 *Mmp8*^−/−^ → WT-CSDS), **k**, splash test (*n* = 6 WT → WT-CON, 7 *Mmp8*^−/−^ → WT-CON, 14 WT → WT-CSDS and 16 *Mmp8*^−/−^ → WT-CSDS), and **l**, elevated plus maze test. Sickness associated behaviours such as **m**, body weight (*n* = 6 WT → WT-CON, 7 *Mmp8*^−/−^ → WT-CON, 15 WT → WT-CSDS and 17 *Mmp8*^−/−^ → WT-CSDS), **n**, food consumption (*n* = 6 WT → WT-CON, 7 *Mmp8*^−/−^ → WT-CON, 15 WT → WT-CSDS and 18 *Mmp8*^−/−^ → WT-CSDS) or **o**, locomotion (*n* = 6 WT → WT-CON, 7 *Mmp8*^−/−^ → WT-CON, 15 WT → WT-CSDS and 17 *Mmp8*^−/−^ → WT-CSDS). (Two-way ANOVA followed by Tukey’s *post hoc* testing [**for all panels**]). Data are shown as mean ± s.e.m. Detailed statistics are in Supplementary Table 2. Source Data

**Extended Data Fig. 9:**
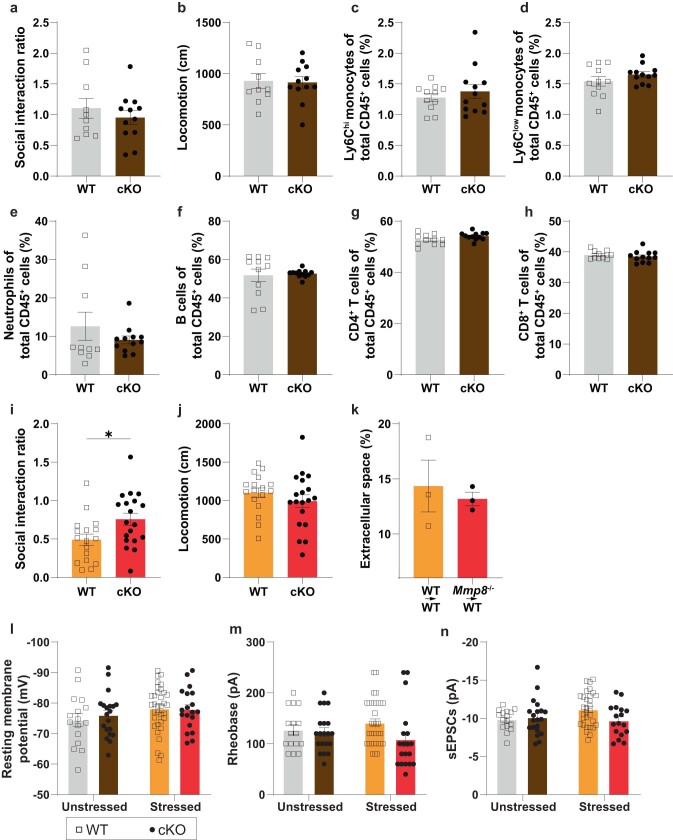
Behavior, leukocyte frequencies and electrophysiology in constitutive *Mmp8* knockout (cKO) mice. **a**, Social interaction ratio (*n* = 10 WT and 12 cKO) **b**, locomotion (*n* = 10 WT and 12 cKO), **c**, Ly6C^hi^ monocytes (*n* = 11 WT and 12 cKO), **d**, Ly6C^low^ monocytes (*n* = 11 WT and 12 cKO), **e**, neutrophils (*n* = 10 WT and 12 cKO), **f**, B cells (*n* = 11 WT and 12 cKO), **g**, CD4^+^ T cells (*n* = 11 WT and 12 cKO), **h**, CD8^+^ T cells (*n* = 11 WT and 12 cKO) in unstressed wildtype (WT) compared to *MMP8*^−/−^ cKO mice at baseline. **i**, Social interaction ratio (*n* = 17 WT and 19 cKO) and **j**, locomotion (*n* = 17 WT and 19 cKO) in wildtype (WT) mice compared to *MMP8*^−/−^ cKO mice after chronic social defeat stress. **k**, Quantification of the extracellular space in prefrontal cortex relative to total brain area in WT compared to *Mmp8*^−/−^ chimeras (*n* = 3 WT- > WT-CSDS, 3 *Mmp8*^−/−^ → WT-CSDS, & 3 WT → WT-CSDS). **l**, Resting membrane potential (*n* = 6 CON-WT, 5 CON-cKO, 5 CSDS-WT and 5 CSDS-cKO; 3 slices per mouse), **m**, rheobase (*n* = 6 CON-WT, 5 CON-cKO, 5 CSDS-WT and 5 CSDS-cKO; 3 slices per mouse) and **n**, amplitude of spontaneous excitatory postsynaptic potentials (sEPSCs) (*n* = 6 CON-WT, 5 CON-cKO, 5 CSDS-WT and 5 CSDS-cKO; 3 slices per mouse). (two-sided Student’s t-test [**for panels a**-**k**]). Two-way ANOVA followed by Tukey’s *post hoc* testing [**for panels l**-**n**]). Data are shown as mean ± s.e.m. Detailed statistics are in Supplementary Table 2. Source Data

To selectively deplete *Mmp8* in peripheral leukocytes and to prevent non-specific developmental effects of germline deletion of *Mmp8*, we created chimeric mice that lack *Mmp8* specifically in peripheral leukocytes (*Mmp8*^−/−^ → WT) or wild-type controls (WT → WT) by bone marrow transplantation (BMT) with haematopoietic stem cells from *Mmp8*^−/−^ or *Mmp8*^+/+^ (wild-type) donor mice. These chimeric mice were then exposed to CSDS and underwent behavioural testing and assessment of the ECS in the NAc and PFC (Fig. 4f). We first validated the efficiency of the BMT experiment and found a complete depletion of MMP8 in the blood of *Mmp8*^*−/−*^ → WT mice, which validated the efficiency of the BMT and confirmed that the source of MMP8 is indeed from peripheral leukocytes (Fig. 4g). We also found high chimerism (85–90%) and no differences in frequencies of peripheral monocytes, neutrophils, cytokines or chemokines between the wild-type and knockout BMT mice (Extended Data Fig. 8a–i), suggesting that peripheral depletion of MMP8 does not lead to major changes in the peripheral immune system. Behaviourally, mice that were transplanted with haematopoietic stem cells from *Mmp8*^−/−^ mice showed less social avoidance towards a CD-1 mouse following CSDS compared with WT → WT mice, as measured by SI ratio and time the experimental mouse spent in the corner (Fig. 4h–j). Similar effects were observed when mice were tested for social interaction with a same-sex juvenile mouse (Fig. 4k). Notably, we did not observe any effects of MMP8 depletion on changes in other non-social stress-related behaviours such as sucrose preference test, splash test or elevated plus maze (Extended Data Fig. 8j–l). We also did not observe differences in sickness-related behaviours such as body weight, food consumption or general locomotion (Extended Data Fig. 8m–o). Furthermore, unstressed *Mmp8* constitutive-knockout (cKO) mice showed no differences in locomotion, social interaction, or leukocyte subpopulation frequencies at baseline (Extended Data Fig. 9a–h) but, in line with data from *Mmp8*^−/−^ bone marrow chimeras, they showed a higher SI ratio indicating less social avoidance compared with age-matched wild-type littermates following CSDS (Extended Data Fig. 9i,j).

## MMP8 regulates ECS and neurophysiology

Finally, we sought to assess whether MMP8 is linked to changes in ECS and NAc neurophysiology. Again, using transmission electron microscopy we assessed the volume of ECS in stressed mice in the NAc and PFC. We found that, compared with WT → WT mice, *Mmp8*^−/−^ → WT mice had reduced ECS in the NAc (Fig. 4l) but no changes in the PFC (Extended Data Fig. 9k). Given that changes in brain ECS and MMP-mediated reorganization of the ECM have been previously associated with altered neuronal physiology^17,53–55^, we performed ex vivo whole-cell patch clamp recordings of medium spiny neurons (MSNs) from the NAc of cKO and wild-type mice (Fig. 4m). Previous studies have shown that CSDS leads to neurophysiological changes in NAc MSNs of SUS but not RES mice, including increased intrinsic neuronal excitability and increased frequency of excitatory postsynaptic currents^56,57^ (EPSCs). In line with our hypothesis, *Mmp8* depletion attenuated stress-induced increased neuronal excitability (Fig. 4n,o) and spontaneous EPSCs (Fig. 4p,q), without affecting the resting membrane potential, rheobase or the amplitude of spontaneous EPSCs (Extended Data Fig. 9l–n).

**Extended Data Fig. 10:**
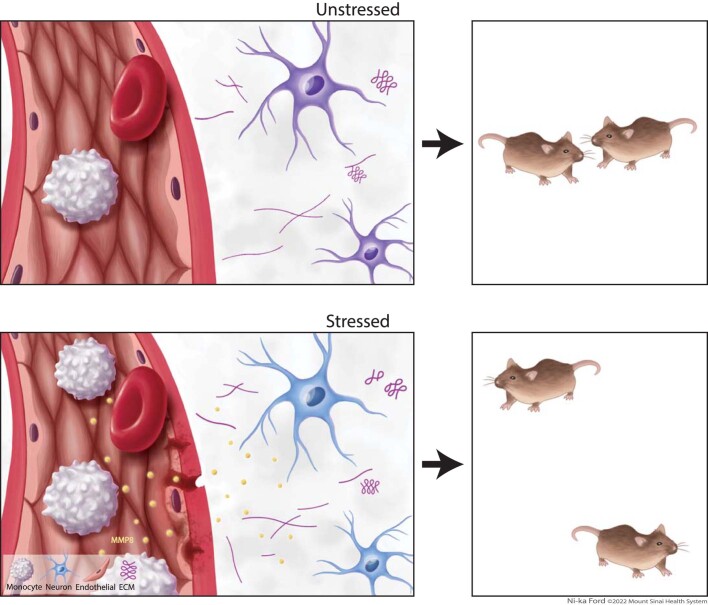
Graphical abstract. Stress-induced increase in peripheral MMP8 leads to social avoidance behaviour associated with alterations in the extracellular space and neurophysiological changes in the nucleus accumbens.

Here, we have shown that stress-induced increases in peripheral MMP8 lead to alterations in the ECS of the NAc associated with altered NAc neurophysiology and social avoidance (Extended Data Fig. 10). We thus provide evidence for a mechanism by which the peripheral immune system can affect neuronal function and behaviour. Although several studies have causally linked peripheral immune factors such as cytokines or different cell types to behavioural alterations, they have shown or hypothesized mechanisms that affect neurons directly—for example, by binding of cytokines to receptors expressed on neurons^58–60^. Here we demonstrate a distinct way in which stress promotes peripheral immune cell interactions with the brain to control social behaviour—that is, via immune cell-derived MMPs from circulation that affect neuronal function by potentially changing the ECS. Further studies are needed to more specifically manipulate different components of the ECS in various brain regions, link them with specific behavioural and neurophysiological changes, and to identify additional peripheral and central factors that affect ECS homeostasis. In addition, a question that needs to be addressed in future studies is the extent to which region-specific monocyte trafficking enables local delivery of secreted factors such as MMP8. Finally, MMP8 depletion prevented social avoidance behaviour after CSDS, with no effect on non-social behaviours. Further research is needed to disentangle the neuroimmune mechanisms of stress-induced social versus non-social behavioural alterations. Together, these data provide important insights into the emerging role of neuroimmune mechanisms in neuropsychiatric disorders, highlighting new peripheral targets for advanced biomarkers and treatment options.

## Methods

### Mice

The following mouse strains were used: For standard CSDS experiments, 7-week-old C57BL/6 J (stock no. 000664) mice were purchased from The Jackson Laboratory. For bone marrow transplantation experiments, 4 week-old B6.SJL-*Ptprc*^*a*^ *Pepc*^*b*^/BoyJ (stock no. 002014, B6 CD45.1) mice were obtained from The Jackson Laboratory. B6.129(Cg)-*Ccr2*^*tm2.1Ifc*^/J (stock no. 017586, *Ccr2*^*rfp*^) and B6.129×1-*Mmp8*^*tm1Otin*^/J (stock no. 005514, *Mmp8*^−/−^) were bred inhouse. Four- to six-month-old male retired CD-1 breeders (Charles River Laboratories, Crl:CD1[ICR]) were used as aggressors for male CSDS. For the female CSDS experiment, male B6N.129S6(Cg)-*Esr1*^*tm1.1(cre)And*^/J (stock no. 017911, *ERa*-*cre* (ERa is also known as *Esr1*)) mice were purchased from The Jackson Laboratory and were crossed with CD-1 females to obtain F_1_ males, which were used as aggressors. Mice purchased from external vendors were allowed to habituate to the animal facility for at least one week. Mice were maintained on a 12 h light:dark cycle (lights on at 07:00, lights off at 19:00) with ad libitum access to food and water. For all behavioural tests, mice were allowed to acclimate to the testing room for at least 1 h. All procedures were performed in accordance with the National Institutes of Health Guide for Care and Use of Laboratory Animals and the Icahn School of Medicine at Mount Sinai (ISMMS) Institutional Animal Care and Use Committee.

### CSDS and SI test

For male CSDS^19^, retired male CD-1 breeders (age: 4–6 months) were used as aggressors. Before each defeat, aggressors were screened for aggressive behaviour for three consecutive days based on previously described criteria^19^. Two days before the start of the defeat, CD-1 mice were housed on one side of a perforated Plexiglass partition. During 10 consecutive days of CSDS, experimental mice (7–8 weeks old) were subjected to direct physical interaction with a CD-1 for 10 min per day (5 min for bone marrow chimera cohorts), and the rest of the day placed on the other side of the Plexiglass divider, allowing for sensory but not direct physical contact. Male aggressors for female CSDS^61,62^ were generated as follows: Heterozygous *ERa-cre* mice were bilaterally injected with a Cre-dependent AAV-DIO-hM3D(Gq)-DREADD (Addgene, 44361-AAV2) into the ventrolateral subdivision of the ventromedial hypothalamus. To activate ERα^+^ cells, intraperitoneal injections of 1.0 mg kg^−1^ clozapine-*N*-oxide (Tocris, 4936) were administered 30 min before each defeat bout. Unstressed control mice were pair-housed across a Plexiglass partition. After the last day of defeat, stressed and unstressed control mice were singly housed (males) or kept in pairs (females). All stressed mice were carefully examined for wounding during the CSDS experiments and mice with excessive wounding were excluded.

### Subthreshold stress

Subthreshold stress is a variation of the CSDS paradigm that is used to unravel pro-susceptible factors^32^ without eliciting behavioural alterations in unmanipulated mice. Experimental mice were exposed to 3× 5 min periods of direct physical interactions with an aggressive CD-1 mouse with a 15 min interval between defeats. 24 h after the last defeat bout, the SI test was conducted as described below.

### SI test

The SI test was performed 24 h after the last defeat session under red-light conditions. After a 1 h habituation period to the behavioural suite, mice were placed into a Plexiglass arena (42 cm × 42 cm × 42 cm, Nationwide Plastics) with a small meshed enclosure on one end. For the first 2.5 min, the experimental mouse freely explored the arena. The mouse was then removed from the arena which was subsequently cleaned with 70 % ethanol, then, a novel social target (CD-1 for males and *ERa*-*cre* for female CSDS) was placed into the enclosure and the experimental mouse was placed back into the arena for another 2.5 min. Locomotor activity was tracked and recorded using a Noldus Ethovision System (Noldus Information Technology, version 11.0). SI ratio was calculated as the ratio between the time the experimental mouse spent in the vicinity of the enclosure (SI zone) when a target mouse was present versus absent. Mice with an SI ratio of ≥1 show a behavioural profile similar to unstressed control mice and were termed resilient, while mice with an SI ratio <1 were termed susceptible. To test social avoidance behaviour towards a juvenile mouse, SI test was performed as described above with a four- to six-week-old male juvenile mouse as a social target. Additional parameters that were measured were total locomotion and time spent in corners, calculated as the sum between the two corners opposite the wire enclosure.

### Chronic variable stress

Chronic variable stress was conducted in female mice as previously described^63^. For 21 days, mice were exposed to daily 1 h long stressors, consisting of either 100 mild foot shocks (0.45 mA), restraint stress in a 50 ml Falcon tube, or tail suspension. For the duration of the stress, mice were group housed.

### Intraperitoneal injection of rMMP8

Before injection, rMMP8 (Bio-techne, 2904-MP-010) was activated ex vivo for 1 h at 37 °C with 1 mM 4-aminophenylmercuric acetate (APMA) in mercury-containing assay buffer (Anaspec, AS-71154) and then diluted in 0.9% sterile saline solution (VWR, 101448-952). For the dose–response experiment, we injected three different doses 50, 100 and 200 µg kg^−1^, and blood was drawn 20 min after the injection via submandibular bleeding and 18 h post-injection using trunk blood. MMP8 was measured as described below. For the behavioural experiments, mice were injected with 100 µg kg^−1^ rMMP8 or APMA 20 min before the defeat bout.

### Stereotaxic surgeries, viral gene transfer or chronic hyaluronidase infusions

Surgeries were performed as described previously^23^. In brief, 6 week-old C57BL/6 J mice were injected intraperitoneally with a mixture of ketamine hydrochloride (100 mg kg^−1^ body weight) and xylazine (10 mg kg^−1^ body weigh). After anaesthesia was confirmed, mice were placed on a stereotaxic instrument (David Kopf Instruments). For the *Cldn5* knockdown experiment, we bilaterally injected 0.5 μl of virus (1.0 × 10^11^ infectious units per ml) expressing either AAV2/9-shRNA or AAV2/9-shRNA-Cldn5 into the NAc (coordinates from bregma: AP + 1.5 mm; ML ± 0.5 mm; DV − 4.4 mm). After 2 weeks of recovery, mice received doxycycline (2 mg ml^−1^ in drinking water) for another 2 weeks. For the hyaluronidase infusion experiment, 27 G guide cannulae were inserted bilaterally into the NAc (from bregma: AP + 1.5 mm; ML ± 0.5 mm; DV − 4.4 mm) and fixed onto the skull using dental cement (Grip cement; Dentsply). After two weeks of recovery, daily infusions of 5 U of hyaluronidase (Sigma-Aldrich, H1136) or saline were completed once daily for 10 consecutive days. All compounds and viruses were infused at a rate of 0.1 μl min^−1^ and allowed to passively diffuse for 5 min before removing the needles.

### Generation of bone marrow chimeras

Bone marrow chimeras were generated as described^10,28^. To ablate the peripheral immune system of the host mouse, 5-week-old male B6 CD45.1 mice were irradiated with a total of 11 Gy, delivered in two doses of 5.5 Gy, 3–4 h apart (X-rad 320 Irradiator (Precision X-Ray)). Haematopoietic progenitor cells were isolated from the femur/tibia of either *Mmp8*^−/−^ or *Mmp8*^+/+^ male donor mice (12 weeks old). One hour after the second dose of irradiation, 1 × 10^6^ cells were injected retro-orbitally in mice anaesthetized with isoflurane. Host mice were then allowed to recover for a total of six weeks. Mice received antibiotic treatment (0.2% in drinking water) (Neomycin trisulfate, N1876, Sigma) during the first three weeks of recovery. The level of chimerism was assessed using flow cytometry, comparing CD45.1 (host) (mouse anti-CD45.1-PE-Cyanine7, clone A20, Invitrogen, 25-0453-81) and CD45.2 (donor) (mouse anti-CD45.2-BV421, clone 104, BD Bioscience, 562895) leukocytes, and measuring MMP8 in plasma (Abcam, ab206982).

### sCPP

sCPP was performed as described^64^. The experiment was done under red-light conditions after mice were habituated to the CPP room for 1 h. The CPP chamber (Med Associates) consisted of 3 different compartments: a neutral middle part, and two adjacent chambers, each with distinct floors (grid pattern) and walls. On the pre-test day, mice were allowed to explore all three chambers for 20 min and the time spent in each chamber was recorded. Based on these durations, mice were balanced to account for pre-test preferences. During the four consecutive conditioning days, mice were conditioned twice per day: In the morning, mice were placed in one chamber for 15 min with a novel, same-sex juvenile (4 to 5 weeks old) C57BL/6 J mouse (paired chamber). In the afternoon, the experimental mouse was put in the empty opposite chamber for the same amount of time (unpaired chamber). On the testing day, mice were again allowed to freely explore all chambers for 20 min and the time spent in each chamber was automatically recorded (Med Associates).

### Sucrose preference test

Sucrose preference test was performed to assess hedonic behaviour towards a sweet gustatory stimulus^11^. Mice were given access to two water bottles (50 ml conical tubes with sipper tops) for 24 h for habituation. Then, one water bottle was exchanged with a bottle containing 1% sucrose (Sigma, S0389) in drinking water. After 24 h, the bottle positions were swapped to prevent position bias. After another 24 h, sucrose preference was assessed as follows (based on weight of bottles): (sucrose (g)/total fluid (g)) × 100%.

### Splash test

The splash test, a test performed to assess self-care behaviour, was conducted under red-light conditions as described previously^11^. In brief, after 1 h of habituation to the testing room, a 10% sucrose solution was gently sprayed onto the lower back of the mouse. Behaviour was recorded for 5 min, and time spent grooming was scored.

### EPM test

The EPM was conducted to assess anxiety-like behaviours^11^. After 1 h of habituation to the testing room, mice were placed on an elevated cross-shaped maze for 5 min under red-light conditions. The four arms (two arms without and two arms with walls, each arm of the maze measuring 12 × 50 cm) were elevated 1 m above the floor. Behaviour was tracked using a Noldus Ethovision System (Noldus Information Technology, version 11.0). Parameters assessed included time spent in closed arms, open arms and in the centre.

### Mass cytometry

Blood was collected directly into fluorescence-activated cell sorting (FACS) buffer (DPBS (Thermo Fisher Scientific, 14190144) containing 0.5% bovine serum albumin (Sigma-Aldrich, A9647) and 2 mM EDTA (Invitrogen, AM9260G)). Cells were pelleted and red blood cells (RBCs) were lysed using RBC lysis buffers (BD, 555899). Immune cells of the brain were isolated as previously described^25^. In brief, mice were anaesthetized with 10% chloral hydrate and transcardially perfused with ice-cold PBS (0.1 M). Brains were extracted, leptomeninges carefully removed and the brains then cut into small pieces using scissors in a total of 3 ml digestion buffer (RPMI (Thermo Fisher Scientific, 11875093) with 2% fetal bovine serum (Thermo Fisher Scientific, A3840001), 2 mM HEPES (Corning, 25-060-CI) and 0.4 mg ml^−1^ collagenase D (Roche, 12352200)). The cell suspension was then incubated for 30 min at 37 °C. Digestion was stopped by adding EDTA (Invitrogen, AM9260G) to a final concentration of 5 mM. Using blunt 18 G needles (BD, 303129), the cell suspension was gently homogenized, and the homogenate was passed through a 70 μm strainer (pre-wet with PBS) (Miltenyi Biotec, 130-095-823). Cells were pelleted, resuspended in 30% Percoll (Millipore Sigma, GE17-0891-01) and centrifuged for 30 min at 23,500*g* without brakes at 4 °C. The myelin layer was aspirated and the middle layer containing leukocytes was transferred into a conical tube. Cells were then washed and stained for 30 min on ice with a mix of metal-conjugated antibodies (Supplementary Table 1). After antibody staining, cells were incubated with cisplatin for 5 min at room temperature as a viability dye to enable exclusion of dead cells. Cells were then fixed in PBS containing 1.6% formaldehyde and a 1:4,000 dilution of Ir nucleic acid intercalator to label all nucleated cells. Immediately prior to acquisition, cells were washed in PBS, then in distilled water, and finally resuspended in distilled water containing a 1/10 dilution of Equation 4 Element Calibration beads (Fluidigm, SKU 201078). After routine instrument tuning and optimization, the samples were acquired on a CyTOF2 Mass Cytometer equipped with a Super Sampler fluidics system (Victorian Airships). The acquisition rate was <500 events per second. The resulting FCS files were concatenated and normalized using a bead-based normalization algorithm in the CyTOF acquisition software and uploaded to Cytobank (https://mtsinai.cytobank.org/cytobank/; Cytobank, Menlo Park, CA, v7.0). FCS files were manually pre-gated for CD45^+^ events, excluding dead cells, doublets and DNA-negative debris (Extended Data Fig. 1a). Data analysis was performed with Clustergrammer, a web-based tool for visualizing and analysing high-dimensional data (https://github.com/ismms-himc/LegendScreen_CyTOF).

### Fluorescence-activated cell sorting and bulk RNA sequencing of leukocyte subpopulations

For the mouse leukocyte subpopulation sequencing experiment, trunk blood was collected directly into FACS buffer. Samples were centrifuged and RBC lysis was performed (BD, 555899). After washing the cell pellet with ice-cold DPBS, Fc receptor blocking (rat anti-CD16/CD32, clone 2.4G2, BD Biosciences, 553141) was performed on ice for 30 min. Cells were pelleted and washed once. Leukocytes were then stained with the following antibodies (all at 1:400): rat anti-CD11b-PE-Cyanine7 (clone M1/70, BioLegend, 101215), rat anti-Ly6C-PerCP–Cy5.5 (clone HK1.4, BioLegend, 128027), rat anti-Ly6G-PE (clone 1A8, BioLegend, 127607), rat anti-B220-FITC (clone RA3-6B2, BioLegend, 103205) and rat anti-CD90.2-APC (clone 53-2.1, BioLegend, 140312) for 30 min on ice protected from light. After an additional wash, cells were sorted directly into Trizol (Themo Fisher Scientific, 15596026) by a BD FACSAria II cell sorter. Raw flow cytometry data were analysed using FlowJo software (FlowJo LLC, version 10.6.2). Samples were flash frozen on dry ice and stored at −80 °C. RNA was extracted using the RNeasy Micro Kit according to the manufacturer’s instructions (Qiagen, 74004). RNA quality, RNA integrity number (RIN) and RNA concentrations were assessed using Nanodrop (Thermo Fischer Scientific) and Bionalyzer (Agilent, 5067-1513). 500 pg of purified RNA was used for library preparation, which was performed using the SMARTer Stranded Total RNASeq Kit –2 - Pico Input Mammalian (Takara, 634413). Libraries were barcoded for multiplexing. Before sequencing, library quality and concentration were measured using Qubit Fluorometric Quantitation (Thermo Fisher). Libraries were sequenced (2 × 150 bp, paired-end reads configuration, v4 chemistry) on an Illumina HiSeq machine at a minimum of 30 million reads per sample. Sequencing was performed at Genewiz. Raw sequencing reads from the samples were mapped to mm10 using HISAT2 v2.1.0^65^. Counts of reads mapping to genes were obtained using htseq-count v0.12.4 against Ensembl v90 annotation^66^. Differential expression analysis was done using DESeq2 v1.26.0 package^67^. The fold change threshold was set at 2 (that is, log_2_(fold change) > |1|). GO terms were determined using DAVID, version 6.8^68^. Only GO terms with an adjusted *P* value < 0.05 (FDR) and an overall of > 5% (involved genes/total genes) were considered.

### Fluorescence-activated cell sorting and flow cytometry of immune cells in leptomeninges, dura and choroid plexus

Leukocyte subpopulation frequencies from brain border regions were isolated as previously described^69^. Mice were anaesthetized with 10% chloral hydrate and transcardially perfused with ice-cold PBS (0.1 M). Leptomeninges, dura and choroid plexus were carefully dissected on ice. Meninges were digested in RPMI (Thermo Fisher Scientific, 11875093) with 1.4 U ml^−1^ Collagenase VIII (Sigma-Aldrich, C2139) and 1 mg ml^−1^ DNAse 1 (Thermo Fisher Scientific, EN0521) for 15 min at 37 °C. Digested dura and leptomeninges and undigested choroid plexus were passed through a 70 μm cell strainer (pre-wet with PBS) (Miltenyi Biotec, 130-095-823) into a 15 ml conical tube. Cells were pelleted (300*g* for 10 min at 4 °C) and washed once with ice-cold PBS. Cells were then resuspended in FACS buffer, Fc receptor binding was blocked (rat anti-CD16/CD32, clone 2.4G2, BD Biosciences, 553141) and cells were stained with a viability dye (Thermo Fisher Scientific, 65-0865-14) for 30 min. Cells were washed and stained with the following fluorophore-conjugated primary antibodies for 30 min on ice (all dilutions: 1:400): rat anti-CD11b–FITC (clone: M1/70, Invitrogen 11-0112-81), Armenian hamster anti-TCRβ-PerCP–Cy5.5 (clone: H57-597, Invitrogen, 45-5961-80), rat anti-Ly6C–APC (clone: HK1.4, Invitrogen, 17-5932-82), rat anti-Ly6G–eFluor 450 (clone: 1A8-Ly6g, Invitrogen, 48-9668-82), rat anti-CD19–PE (clone: IDE, BD Pharmingen, 553786), rat anti-CD45–PE–Cy7 (clone: 30-F11, BD Pharmingen, 552848). After an additional wash, cells were resuspended in FACS buffer and sorted by a BD FACSAria II using the 70 μm nozzle to sort cells into 1.5 ml Eppendorf tubes containing TRIzol LS with a sort speed of approximately 10,000 events per second. Raw flow cytometry data were analysed using FlowJo software (FlowJo, version 10.8.1).

### FACS and single-cell RNA sequencing

Brain-trafficking monocytes and brain-resident myeloid cells were isolated based on previous published protocols^70^. Twenty-four hours after the SI test, mice were euthanized by injecting 10% chloral hydrate and perfused transcardially with ice-cold 0.1 M PBS (pH 7.4). Brains were rapidly dissected, leptomeninges carefully removed, and brains put in ice-cold PBS (for brain-trafficking monocyte RNA-sequencing experiment) or bilateral NAc tissue punches were obtained from 1 mm thick coronal slices using 1.2 mm punches (for resident myeloid cell RNA-sequencing experiment) (GE Healthcare Life Sciences, 1205×41). All the following steps were performed strictly on ice. For whole brains, tissue was cut into small pieces, for punches no shredding was needed. Tissue was then transferred to DPBS and homogenized with pestles (Sigma, D8938-1) in ice-cold PBS (20 stokes with pestle A, 20 stokes with pestle B). The cell suspension was then passed through a 70 μm cell strainer (pre-wet with PBS) (Miltenyi Biotec, 130-095-823) into a 15 ml conical tube. Cells were pelleted (300*g* for 5 min at 4 °C), resuspended in 10 ml of ice-cold 40% isotonic Percoll (Millipore Sigma, GE17-0891-01) (diluted in PBS) and centrifuged for 30 min at 500*g* at 4 °C with full acceleration and braking. The myelin layer was aspirated, then the cell pellet was washed with 10 ml of ice-cold PBS by centrifuging at 300*g* for 5 min at 4 °C. Cells were then resuspended in FACS buffer, Fc receptor binding was blocked (rat anti-CD16/CD32, clone 2.4G2, BD Biosciences, 553141) and then cells were stained with a viability dye (Thermo Fisher Scientific, 65-0865-14) for 30 min. Cells were washed and stained with a combination of the following fluorophore-conjugated primary antibodies: rat anti-CD45–BV510 (clone 30-F11, BioLegend, 103137), rat anti-CD11b–PerCP-Cyanine5.5 (clone M1/70, BioLegend, 101227), rat anti-Ly6C–APC-Cyanine7 (clone HK1.4, BioLegend, 128025), and rat anti-Ly6G–eFluor 450 (clone 1A8-Ly6g, Thermo Fisher Scientific, 48-9668-82) at a 1:400 dilution for 30 min on ice. After an additional wash, cells were sorted by a BD FACSAria II using the 70 μm nozzle to sort single cells into 96-well plates containing master mix (see below) with a sort speed of approximately 10,000 s^−1^. Raw flow cytometry data were analysed using FlowJo software (FlowJo LLC, version 10.6.2). All single-cell RNA-sequencing experiments were performed at the Single Cell Core Facility of the Sulzberger Columbia Genome Center, New York. Library preparation and RNA sequencing was performed as described previously^71^. In brief, cells were directly sorted into master mix, containing 1× Maxima Reverse Transcriptase Buffer (Thermo Fisher Scientific, EP0742), 40 U Maxima H Minus Reverse Transcriptase (Thermo Fisher Scientific, EP0751), 4 U SuperaseIN (Thermo Fisher Scientific, AM2694), 15% PEG (VWR, 97061-102), 1 μM TSO (Integrated DNA Technologies), and nuclease-free water. Template-switching reverse transcription was performed with adapter-linked oligo primers containing both cell- and molecule-specific barcodes (Supplementary Table 6). Excess primers were removed by adding 2 μl of Exonuclease I (Thermo Fisher Scientific, EN0581) mix to each well and incubated at 37 °C for 30 min, 85 °C for 15 min, 75 °C for 30 s. All wells were then pooled into a single 15 ml conical tube and cDNA was purified and concentrated with Dynabeads MyOne Silane beads (Thermo Fisher Scientific, 37002D). The cDNA was split into duplicate reactions of 25 μl cDNA, 25 μl of 2× HIFI HotStart Ready Mix (Kapa Biosystems, 07958927001), and 0.2 M SMART PCR Primer (Supplementary Table 6). PCR was performed as described above. cDNA was purified with AMPure XP beads (Beckman Coulter, A63880), visualized on an Agilent TapeStation and quantified with a Qubit II fluorometer (Thermo Fisher Scientific). Library preparation was performed using a modified protocol of the Nextera XT kit (Illumina, FC-131-1024), purified twice with AMPure XP beads (Beckman Coulter, A63880), and visualized and quantified as described above. Pooled, 3’-end libraries were sequenced on an Illumina NextSeq 500/550 apparatus. Reads were aligned to the mouse genome reference GRCm38 using STAR (version 2.5)^72^. Reads were assigned to cells and unique molecular identifiers^73^. The expression matrix for single-cell data was processed using the package Seurat v3.1.5 in R^74^. Features for which fewer than 3 cells were detected were removed, effectively excluding unexpressed features. Cells having at least 1,000 and at most 4,000 features were retained. Cells with more than 5% of reads mapping to mitochondrial genes were discarded. The NormalizeData function was used to log-normalize the dataset with a scale factor of 10,000. The top 2,000 most variable features across cells were found using the function FindVariableFeatures. The ScaleData function was applied to scale the dataset. The variable features were used to carry out dimensional reduction using principal components analysis. The optimal number of principal components to be used for dimensional reduction using UMAP was determined using ElbowPlot. FindNeighbors and FindClusters functions were utilized to construct a nearest neighbour graph and cluster cells in the dataset. UMAP was generated using the function DimPlot. The FindAllMarkers function was applied to determine markers for clusters in the UMAP plot. The FindMarkers function was used to carry out differential expression analysis for the three experimental groups.

### iDISCO+ staining, imaging and ClearMap analysis

Twenty-four hours after the SI test, *Ccr*2^*rfp*+/−^ mice were injected with 10% chloral hydrate and transcardially perfused with ice-cold 0.1 M PBS followed by 4% paraformaldehyde (PFA) (Electron Microscopy Sciences, 15713 S). Intact brains were dissected out of the skull and post-fixed in 4% PFA in PBS at 4 °C for 18 h. Brains were then cleared and stained according to the iDISCO+ staining protocol (http://www.idisco.info). The primary rabbit anti-RFP antibody (Rockland, 600-401-379, 1:1,000) and the corresponding secondary antibody (donkey anti-rabbit IgG, Alexa Fluor 647, Thermo Fisher Scientific, A-31573, 1:1,000) were incubated with the brains for 7 days each at 37 °C. A LaVision light-sheet microscope with zoom body was used for sagittal half brain scanning with dynamic focus and a step size of 4 µm. Brain images were processed as previously described using ClearMap (version 1)^31^. RFP^+^ cells were quantified using the cell detection module, with cell detection parameters optimized and validated based on the intensity and shape parameters of the signal. The autofluorescence channel was aligned to the Allen Institute’s Common Coordinate Framework using the Elastix toolbox. Brain areas were collapsed into their parent regions prior to analyses.

### Western blot

Bilateral NAc tissue punches were briefly thawed on ice and digested for 60 min at 37 °C in 20 U ml^−1^ chondroitinase ABC (Sigma-Aldrich, C3667) in 25 mM Tris-buffered saline (Thermo Fisher Scientific, BP2471) with protease inhibitors (Thermo Fisher Scientific, 1861281). Samples were immediately cooled on ice, centrifuged for 20 min at 21,000*g* at 4 °C and the supernatant was transferred to a new tube. Protein concentration was determined using the Pierce BCA Protein Assay Kit (Thermo Fisher Scientific, 23227). Samples were flash frozen and stored at −80 °C. Total protein (20 μg) was separated by electrophoresis with a SDS–PAGE polyacrylamide gel (Bio-Rad, 4561034) and transferred to a PVDF membrane (Bio-Rad, 1704157). The membrane was blocked with 5 % non-fat dry milk (Bio-Rad, 1706404) in 0.1% Tween-20 (Sigma-Aldrich, P7949) in Tris-buffered saline (Thermo Fisher Scientific, BP2471, TBS-T) and incubated overnight with primary antibodies against aggrecan (1:1,000, Sigma-Aldrich, AB1031) or horseradish peroxidase (HRP) conjugated β-actin (1:2,000, Cell Signaling, 12262) in 5% non-fat dried milk (Sigma-Aldrich, A9647) in TBS-T. Membranes were then washed for 1 h with TBS-T and then incubated with secondary antibodies (1:10,000, anti-rabbit IgG HRP-linked, Cell Signaling, 7074) for 3 h at room temperature. After washing the membrane, visualization was performed using Pierce ECL Western Blotting Substrate (Thermo Fisher Scientific, 32209) with an iBright CL1500 Imaging System (Thermo Fisher Scientific, A44114). Quantification was done with ImageJ (NIH, v1.53f51)^75^. Uncropped blots are available in Supplementary File 1.

### Quantitative real-time PCR

RNA from fluorescence-activated cell-sorted monocytes was isolated using the RNeasy Micro Kit according to the manufacturer’s instructions (Qiagen, 74004). RNA quality and RNA concentrations were assessed using Nanodrop (Thermo Fischer Scientific). RNA was reversed transcribed to cDNA using qScript (QuantaBio, 95048-100) and the PCR reaction was performed using the SYBR Fast Advanced Master Mix system (Thermo Fisher Scientific, A46012) with the following primers (Integrated DNA Technologies): *Mmp8* (Mm.PT.58.6942600, primer 1: AGGATCAGTGGAGTGAGAGAG; primer 2: CAAGGTATTGGAGGAGATGCTC); *Gapdh* (Mm.PT.39a.1, primer 1: GTGGAGTCATACTGGAACATGTAG; primer 2: AATGGTGAAGGTCGGTGTG). Gene expression analysis was done using the 2^(–Δ^^Δ*C*T)^ method^76^ and samples were normalized to the housekeeping gene *Gapdh*.

### Ex vivo electrophysiology

Brains were rapidly extracted from isoflurane-anaesthetized mice, and coronal sections (250 µm) were sliced using a Compresstome (VF-210-0Z, Precisionary Instruments) in cold (0–4 °C) sucrose-based artificial cerebrospinal fluid (aCSF) containing: 87 mM NaCl (Sigma-Aldrich, S7653), 2.5 mM KCl (Sigma-Aldrich, P9333), 1.25 mM NaH_2_PO_4_ (Sigma-Aldrich, 71507), 4 mM MgCl_2_ (Sigma-Aldrich, M2670), 0.5 mM CaCl_2_ (Sigma-Aldrich, C8106), 23 mM NaHCO_3_ (Sigma-Aldrich, S6297), 75 mM sucrose (Sigma-Aldrich, S7903), 25 mM glucose (Sigma-Aldrich, G7021). After 60 min in aCSF at 32 °C for recovery, slices were kept in oxygenated (95% O_2_, 5% CO_2_) aCSF containing 130 mM NaCl (Sigma-Aldrich, S7653), 2.5 mM KCl (Sigma-Aldrich, P9333), 1.2 mM NaH_2_PO_4_ (Sigma-Aldrich, 71507), 2.4 mM CaCl_2_ (Sigma-Aldrich, C8106), 1.2 mM MgCl_2_ (Sigma-Aldrich, M2670), 23 mM NaHCO_3_ (Sigma-Aldrich, S6297), 11 mM glucose (Sigma-Aldrich, G7021) at room temperature for the rest of the day and individually transferred to a recording chamber continuously perfused at 2 to 3 ml min^−1^ with oxygenated aCSF. Patch pipettes (4–6 MΩ) were pulled from thin wall borosilicate glass using a micropipette puller (P-97, Sutter Instruments) and filled with a potassium gluconate-based intra-pipette solution containing: 116 mM KGlu (Sigma-Aldrich, P1847), 20 mM HEPES (Sigma-Aldrich, H3375), 0.5 mM EGTA (Sigma-Aldrich, E0396), 6 mM KCl (Sigma-Aldrich, P9333), 2 mM NaCl (Sigma-Aldrich, S7653), 4 mM ATP (Sigma-Aldrich, A9187), 0.3 mM GTP (Sigma-Aldrich, 51120) (pH adjusted to 7.2 and osmolarity to 290 mOsm). Cells were visualized using an upright microscope with an IR-DIC lens and illuminated with a white light source (Scientifica). Excitability was measured in current-clamp mode by injecting incremental steps of current (0–300 pA, +20 pA at each step). For recording of sEPSCs, NAc MSNs were recorded from in voltage-clamp mode at −70 mV. Whole-cell recordings were performed using a patch clamp amplifier (Axoclamp 200B, Molecular Devices) connected to a Digidata 1550 LowNoise acquisition system (Molecular Devices). Signals were low pass filtered (Bessel, 2 kHz) and collected at 10 kHz using the data acquisition software pClamp 11 (Molecular Devices). Electrophysiological recordings were extracted using Clampfit 11 (Molecular Devices) and analysed with R (version: 3.6.1, http://www.R-project.org). All groups were counterbalanced by days after CSDS. All recordings were performed while blinded to the experimental conditions.

### Human participants and processing of biospecimen

Study participants with MDD and healthy controls, as assessed according to SCID-5^77^, were recruited through the Depression and Anxiety Center for Discovery and Treatment at the ISMMS. The ISMMS review board approved the study, and written informed consent was obtained from all participants prior to any study procedure. Participants were compensated for their time and effort. They provided demographic information and underwent a psychiatric evaluation using the SCID-5 conducted by trained study staff. Participants completed the Quick Inventory of Depressive Symptomatology-SR (QIDS-SR) to measure depressive symptom severity^78^. The Perceived Stress Scale^27^, a 10-item self-rating scale, was used to determine perceived stress levels. All participants underwent biochemistry and haematological laboratory testing, urine toxicology and pregnancy (if applicable) testing. At the time of enrolment, all participants were free of medications known to affect the immune system for at least two weeks. Participants were free of active infections or systemic illness. Subjects with concomitant unstable medical illnesses were excluded. Participants were free of current substances of abuse. On the day of blood draw, patients were fasted for at least 6 h. Blood was drawn into Vacutainer Gold Top 5 ml Silica Gel tubes (BD, 365968) for serum isolation, EDTA tubes (BD, 365975) to assess complete blood count and differential count (Sysmex XN-9100 Automated Hematology System) and into BD Vacutainer CPT tubes (BD, 362761) for the isolation of peripheral blood mononuclear cells (PBMCs). For serum, blood was allowed to clot for >30 min, then centrifuged at 1,300*g* for 15 min at 4 °C, then aliquoted and stored at −80 °C. PBMCs were isolated according to the manufacturer’s instructions and cryopreserved in liquid nitrogen. On the day of analysis, all PBMCs were carefully thawed in a water bath at 37 °C. Cells were pelleted (300*g* for 10 min at 4 °C) and washed once with ice-cold PBS. Cells were then resuspended in FACS buffer. Fc receptor binding was blocked using anti-CD16/32 (clone 2.4G2, Bio X Cell, BE0307) and cells were stained with a viability dye (Thermo Fisher Scientific, 65-0865-14) for 30 min. Cells were washed and stained with the following fluorophore-conjugated primary antibodies for 30 min on ice (all dilutions: 1:400): mouse anti-CD45–V500 (clone HI30, Fisher Scientific, BDB560779), mouse anti-CD19–PE–Cy7 (clone SJ25C1, Fisher Scientific, BDB560911), mouse anti-CD24–PE (clone ML5, Fisher Scientific, BDB560991), mouse anti-CD27–APC (clone L128), mouse anti-CD38 PerCP–Cy5.5 (clone HIT2, Fisher Scientific, BDB551400) and mouse anti-IgD–V450 (clone IA6-2, Fisher Scientific, BDB561309). Cells were washed, then resuspended in FACS buffer before acquisition on a BD LSRFortessa cell analyser (BD Biosciences). Flow cytometry data were acquired using FACS Diva software (BD, v.9). Data were analysed using FlowJo software (FlowJo LLC, version 10.8.1). Gating of B cell subtypes was performed as described^79^.

### Enzyme-linked immunosorbent assay

Enzyme-linked immunosorbent assays were performed according to the manufacturer’s instructions (mouse MMP8: Abcam, ab206982; human MMP8: R&D Systems, DMP800B). For brain lysates, total protein was measured with the Pierce BCA Protein Assay Kit (Thermo Fisher Scientific, 23225). Plates were read on a SpectraMax 340PC384 microplate reader (Molecular Devices) and MMP8 or total protein levels were calculated from a serial dilution curve using SoftMax Pro 5 software (Molecular Devices).

### Multiplex assays

Mouse plasma cytokines and chemokines were determined with the Milliplex MAP mouse cytokine/chemokine magnetic bead panel multiplex assay according to the manufacturer’s instructions (Millipore Sigma, MCYTOMAG-70K), and mouse MMP2, MMP3, proMMP9 and MMP12 were measured with Milliplex MAP Mouse MMP Magnetic Bead Panels 1 and 2 (Millipore Sigma, MMMP1MAG-79K, MMMP2MAG-79K).

### Transmission electron microscopy and image analysis

Mice were injected with 10% chloral hydrate and transcardially perfused with 0.1 M sodium cacodylate buffer followed by ice-cold 2% PFA and post-fixed with 0.5% PFA at 4 °C. Tissue was sectioned on a vibratome, and freeze substitution and low temperature embedding of the specimens was performed as described^80–82^. Slices were cryoprotected by immersion in increasing concentrations of glycerol (from 10% to 30% in PBS) (v/v). Sections were plunged rapidly into liquid propane cooled by liquid nitrogen (−190 °C) in a Universal Cryofixation System KF80 (Reichert-Jung). The samples were immersed in 1.5% uranyl acetate dissolved in anhydrous methanol (−90 °C, 24 h) in a cryosubstitution AFS unit (Leica). The temperature was raised from −90 °C to −45 °C in steps of 4 °C/h. After washing with anhydrous methanol, the samples were infiltrated with Lowicryl HM20 resin (Electron Microscopy Sciences) at −45 °C. Polymerization with ultraviolet light (360 nm) was performed for 48 h at −45 °C, followed by 24 h at 0 °C. Ultrathin sections (80 nm) were cut with a diamond knife on a Leica UC7 ultramicrotome and mounted on 300 mesh copper grids using a Coat-Quick adhesive pen (Electron Microscopy Sciences). Images (*n* = 10 per mouse) were taken using a Hitachi 7700 electron microscope (Hitachi High-Technologies Corporation America) equipped with a XR81-B-M1-BT-FX, 8 Megapixel digital camera (Advanced Microscopy Techniques). Images were then imported into Adobe Photoshop (Adobe, 2022) and the ECS was manually scored using a computer tablet. Scoring was done by two independent investigators blinded to experimental conditions. Images were then imported into ImageJ (v1.53f51)^75^ and the percentage of marked area/total area was calculated.

### Immunohistochemistry and confocal microscopy

Mice were injected with 10% chloral hydrate and transcardially perfused with ice-cold 0.1 M PBS (pH 7.4) followed by ice-cold 4% PFA (Electron Microscopy Sciences, 15713 S). Intact brains were dissected out of the skull and post-fixed in 4 % PFA at 4 °C for 18 h. Brains were then cryoprotected in 30% sucrose (Sigma, S0389), frozen and sliced on a cryostat at 35 μm thickness. Sections were washed with PBS three times and incubated in blocking solution (3% normal donkey serum (Jackson Immuno Research, 017-000-121), 0.3% Triton X-100 (Sigma, T9284) in PBS) for 2 h. Sections were then incubated in primary antibodies (rat anti-CD31, 1:300, Biolegend, 102501; rabbit anti-RFP, 1:300, Rockland, 600-401-379; goat anti-RFP, 1:200, Rockland, 200-101-379; rabbit; rabbit anti-AQP4, 1:400, Thermo Fisher Scientific, PA5-85767) overnight at 4 °C. The next day, sections were washed in PBS with 0.3% Tween-20 (PBST (Sigma, P7949)) three times for 15 min each, then incubated with anti-rabbit-Cy2 and anti-rat-Cy5 secondary antibodies for 2 h (1:400, Jackson Immuno Research, 711-225-152 and 712-175-153, respectively). Sections were washed again three times with PBST. Slices were then mounted on slides, air-dried overnight, dehydrated, and coverslipped with DPX (Electron Microscopy Sciences, 13510). All slices were imaged using a Zeiss LSM 780 confocal microscope. 3D reconstruction was performed with the IMARIS software (Oxford Instruments, v9.9).

### Biotinylation

Biotinylation of mouse rMMP8 (Bio-techne, 2904-MP-010) was performed using the EZ-Link Sulfo-NHS-Biotin kit according to the manufacturer’s instructions (Thermo Fisher Scientific, A39256). Biotinylated rMMP8 was separated from unbound biotin using Pierce C18 Spin Columns, 7 K MWCO, (Thermo Fisher Scientific, 89882), which recovers proteins and macromolecules larger than 7 kDa. Biotinylated rMMP8 was injected retro-orbitally into anaesthetized mice. After 2 h of circulation, mice were euthanized and perfused with ice-cold PBS followed by 4% PFA. Brain tissue processing and imaging was performed as described in the Immunohistochemistry and confocal microscopy section, with the following antibodies: Biotin was visualized using the Oregon Green 488 conjugate of NeutrAvidin biotin-binding protein (Thermo Fisher Scientific, A6374). Counterstaining was performed using rabbit anti-NeuN (1:500, Abcam, ab177487) and rat anti-CD31 (1:300, Biolegend, 102501).

### Statistical analysis

Detailed statistical information for each experiment can be found in Supplementary Table 2. Unless described otherwise, statistical analyses were performed with GraphPad Prism software (GraphPad Software, version 9) or SPSS version 24 (IBM, SPSS). Outliers were identified using the Grubbs’ test and excluded from statistical analyses. Level of statistical significance was set at *P* < 0.05.

### Reporting summary

Further information on research design is available in the Nature Portfolio Reporting Summary linked to this article.

## Online content

Any methods, additional references, Nature Portfolio reporting summaries, source data, extended data, supplementary information, acknowledgements, peer review information; details of author contributions and competing interests; and statements of data and code availability are available at 10.1038/s41586-023-07015-2.

### Supplementary information

Supplementary File 1 This file contains the uncropped western blots.

Reporting Summary

Supplementary Table 1 This file contains the antibodies used in the CyTOF experiments.

Supplementary Table 2 This file contains detailed statistical information.

Supplementary Table 3 This file contains the sociodemographic variables and clinical data of the individuals shown in Fig. 1d–g.

Supplementary Table 4 This file shows the correlations between monocytes in specific brain regions and SI ratio.

Supplementary Table 5 This file contains the sociodemographic variables and clinical data of the individuals shown in Fig. 3c,d.

Supplementary Table 6 This file shows the primer sequences used in the single-cell RNA-sequencing experiments.

### Source data

Source Data Fig. 1

Source Data Fig. 2

Source Data Fig. 3

Source Data Fig. 4

Source Data Extended Data Fig. 1

Source Data Extended Data Fig. 2

Source Data Extended Data Fig. 3

Source Data Extended Data Fig. 4

Source Data Extended Data Fig. 5

Source Data Extended Data Fig. 6

Source Data Extended Data Fig. 7

Source Data Extended Data Fig. 8

Source Data Extended Data Fig. 9

## Data Availability

RNA-sequencing data have been deposited in the Gene Expression Omnibus under accession number GSE202662 (token: wvwdoiicjjcjbyt). Source data are provided with this paper.
