# Supplementary material for: Circulating myeloid-derived MMP8 in stress susceptibility and depression
Source: Nature. 2024 Feb 7;626(8001):1108–15. doi: 10.1038/s41586-023-07015-2 (PMC10901735; doi:10.1038/s41586-023-07015-2)

nature portfolio

https://doi.org/10.1038/s41586-023-07015-2

Supplementary information

Circulating myeloid-derived MMP8 in stress susceptibility and depression

In the format provided by the authors and unedited

Aggrecan

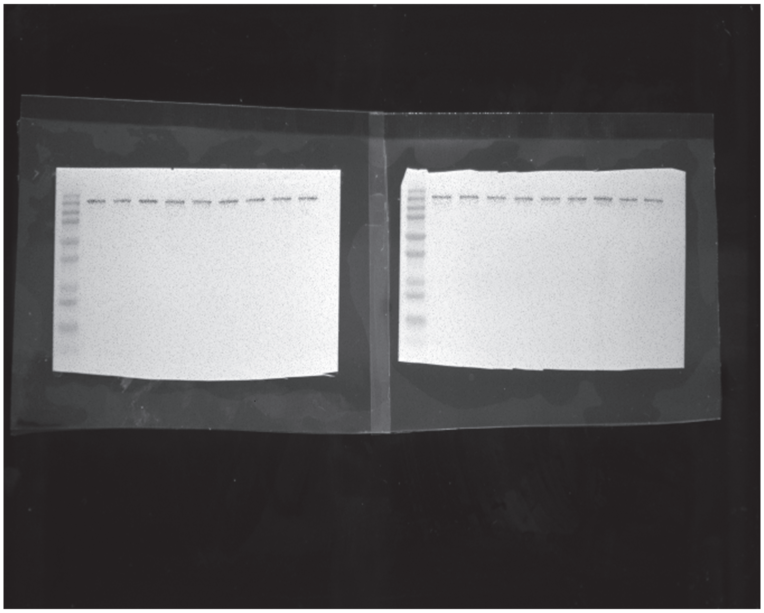

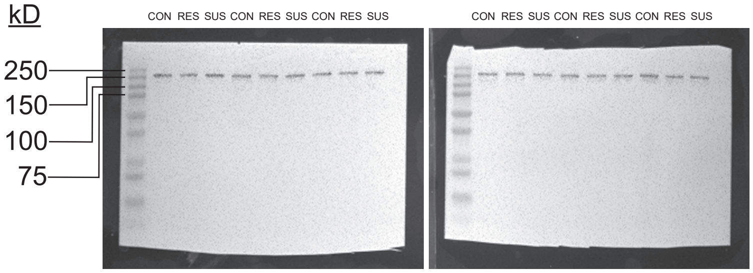

β-Actin

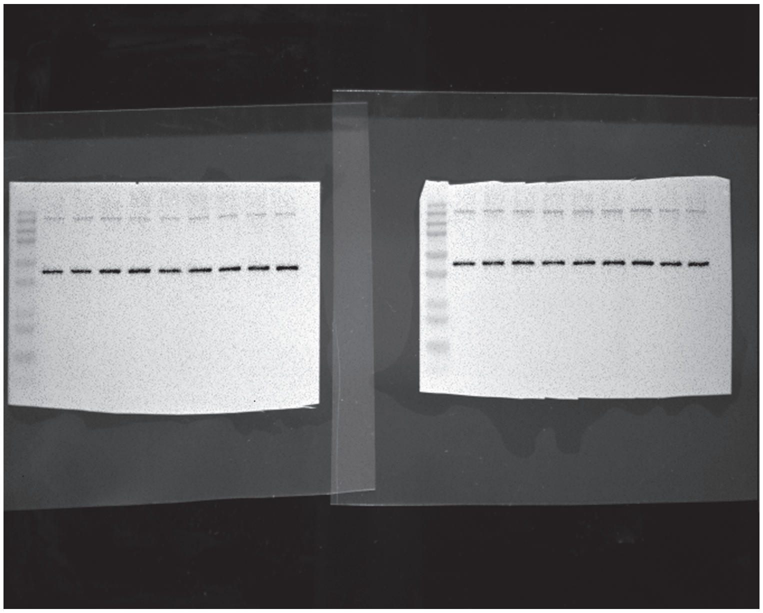

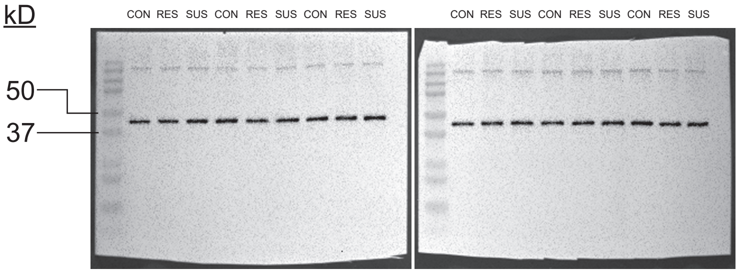

Supplement: Supplementary file 1 — This file contains the uncropped western blots. [file 41586_2023_7015_MOESM1_ESM.pdf]
