# Supplementary material for: Circulating myeloid-derived MMP8 in stress susceptibility and depression
Source: Nature. 2024 Feb 7;626(8001):1108–15. doi: 10.1038/s41586-023-07015-2 (PMC10901735; doi:10.1038/s41586-023-07015-2)
Supplement: Supplementary file 2 — Reporting Summary [file 41586_2023_7015_MOESM2_ESM.pdf]

nature portfolio

Corresponding author(s): Scott J. Russo

Last updated by author(s): 05/11/2022

Reporting Summary

Nature Portfolio wishes to improve the reproducibility of the work that we publish. This form provides structure for consistency and transparency in reporting. For further information on Nature Portfolio policies, see our Editorial Policies and the Editorial Policy Checklist.

Statistics

For all statistical analyses, confirm that the following items are present in the figure legend, table legend, main text, or Methods section.

n/a	Confirmed	
☐	☒	The exact sample size (*n*) for each experimental group/condition, given as a discrete number and unit of measurement
☐	☒	A statement on whether measurements were taken from distinct samples or whether the same sample was measured repeatedly
☐	☒	The statistical test(s) used AND whether they are one- or two-sided<br>*Only common tests should be described solely by name; describe more complex techniques in the Methods section.*
☐	☒	A description of all covariates tested
☐	☒	A description of any assumptions or corrections, such as tests of normality and adjustment for multiple comparisons
☐	☒	A full description of the statistical parameters including central tendency (e.g. means) or other basic estimates (e.g. regression coefficient) AND variation (e.g. standard deviation) or associated estimates of uncertainty (e.g. confidence intervals)
☐	☒	For null hypothesis testing, the test statistic (e.g. *F*, *t*, *r*) with confidence intervals, effect sizes, degrees of freedom and *P* value noted<br>*Give P values as exact values whenever suitable.*
☒	☐	For Bayesian analysis, information on the choice of priors and Markov chain Monte Carlo settings
☒	☐	For hierarchical and complex designs, identification of the appropriate level for tests and full reporting of outcomes
☐	☒	Estimates of effect sizes (e.g. Cohen's *d*, Pearson's *r*), indicating how they were calculated

Our web collection on statistics for biologists contains articles on many of the points above.

Software and code

Policy information about availability of computer code

Data collection	The following softwares were used for data collection:<br>- Noldus Ethovision System (Noldus Information Technology Inc, Version 11.0, Leesburg, VA)<br>- pClamp 11 (Molecular Devices)<br>- SoftMax Pro 5 software (Molecular Devices)

Data analysis

The following softwares were used for data analysis:
- FlowJo (FlowJo LLC, Version 10.6.2)
- Cytobank (https://mtsinai.cytobank.org/cytobank/; Cytobank, Menlo Park, CA, 7.0)
- Clustergrammer (https://github.com/ismms-himc/LegendScreen_CyTOF)
- HISAT2 (v2.1.0)
- Htseq-count (v0.12.4)
- DESeq2 (v1.26.0)
- Database for Annotation, Visualization, and Integrated Discovery (DAVID) (https://david-d.ncifcrf.gov/)
- Seurat (v3.1.5)
- Clearmap (Version 1)
- Adobe Photoshop (Adobe, 2022)
- ImageJ (v1.53f51)
- IMARIS (Version 9.9, Oxford Instruments Group)
- GraphPad Prism software (Version 9, GraphPad Software Inc.)
- SPSS version 24 (IBM Corp., SPSS Inc., Chicago IL, USA).

For manuscripts utilizing custom algorithms or software that are central to the research but not yet described in published literature, software must be made available to editors and reviewers. We strongly encourage code deposition in a community repository (e.g. GitHub). See the Nature Portfolio guidelines for submitting code & software for further information.

Data

Policy information about availability of data

All manuscripts must include a data availability statement. This statement should provide the following information, where applicable:
- Accession codes, unique identifiers, or web links for publicly available datasets
- A description of any restrictions on data availability
- For clinical datasets or third party data, please ensure that the statement adheres to our policy

RNA-seq data have been deposited in the Gene Expression Omnibus under accession number (GSE ID: GSE202662, reviewer token: wvwdoiicjjcjbyt). All the other data are available from the corresponding author upon reasonable request.

Field-specific reporting

Please select the one below that is the best fit for your research. If you are not sure, read the appropriate sections before making your selection.

☒ Life sciences ☐ Behavioural & social sciences ☐ Ecological, evolutionary & environmental sciences

For a reference copy of the document with all sections, see nature.com/documents/nr-reporting-summary-flat.pdf

Life sciences study design

All studies must disclose on these points even when the disclosure is negative.

Sample size	We did not use statistical methods to pre-determine sample sizes. Sample sizes were chosen according to previous experiments (Hodes et al., 2014; Menard et al., 2017; Pfau et al., 2019).
Data exclusions	Single-cell RNA-sequencing: The following cells were excluded from the single-cell RNA-seq data analysis: Cells with less than 1,000 features, more than 4,000 features or more than 5% of reads mapping to mitochondrial genes.<br>Grubb's test was used to exclude outliers from the behavioural experiments. This test was only performed once per data set.
Replication	Data was collected using biological replicates. All major mouse findings of the manuscript (except RNA-sequencing experiments) were replicated in at least two different cohorts.
Randomization	Mice were assigned to their groups (stress-susceptible or resilient) based on their social interaction ratio.
Blinding	While experimenters were not blind to group allocation for data collection (except electrophysiology experiments), analyses (scoring of behavioural videos, scoring of transmission electron microscopy experiments, scoring of immunohistochemical images) were performed blind to experimental conditions.

Reporting for specific materials, systems and methods

We require information from authors about some types of materials, experimental systems and methods used in many studies. Here, indicate whether each material, system or method listed is relevant to your study. If you are not sure if a list item applies to your research, read the appropriate section before selecting a response.

Materials & experimental systems

n/a	Involved in the study
☐	☒ Antibodies
☒	☐ Eukaryotic cell lines
☒	☐ Palaeontology and archaeology
☐	☒ Animals and other organisms
☐	☒ Human research participants
☒	☐ Clinical data
☒	☐ Dual use research of concern

Methods

n/a	Involved in the study
☒	☐ ChIP-seq
☐	☒ Flow cytometry
☒	☐ MRI-based neuroimaging

Antibodies

Antibodies used

Blood CyTOF: Rh103Di::Viability (Fluidigm, #201103A), In113Di::Thy1.2 (clone 30-H12, Biolegend, #105302), Pr141Di::Ly6G (clone 1A8, Fluidigm, #3141008B), Nd142Di::CD11c (clone N418, Biolegend, #117302), Nd143Di::TCRb (clone H57-597, Fluidigm, #3143010B), Nd144Di:CD24 (clone M1/69, Biolegend, #101802), Nd145Di::CD69 (clone H1.2F3, Fluidigm, #3145005B), Nd146Di::F4/80 (clone BM8, Fluidigm, #3146008B), Sm147Di::CD45 (clone 30-F11, Fluidigm, #3147003B), Nd148Di::CD11b (clone M1/70, Biolegend, #101202), Sm149Di::CD19 (clone 6D5, Biolegend, #115502), Nd150Di::IgD (clone 11-26c.2a, Biolegend, #405702), Eu151Di::CD25 (clone 3C7, Fluidigm, #3151007B), Sm152Di::SiglecF (clone S17007L, Biolegend, #155502), Sm154Di::CD169 (clone 3D6.112, Biolegend, #142402), Gd156Di::CD64 (clone X54-5/7.1, Biolegend, #139302), Gd160Di::CD62L (clone MEL-14, Fluidigm , #3160008B), Dy161Di::CD103 (clone 2E7, Biolegend, #121402), Dy162Di::Ly6C (clone HK1.4, Biolegend, #128002), Erl166Di::CD117 (clone 2B8, Biolegend, #105802), Er168Di::CD8 (clone 53-6.7, Biolegend, #100702), Tm169Di::CD45 (clone 30-F11, Fluidigm, #3147003B), Er170Di::NK1.1 (clone PK136, Fluidigm, #3170002B), Yb171Di::CD44 (clone IM7, Biolegend, #103002), Yb172Di::CD4 (clone RM4-5, Fluidigm, #3172003B), Yb174Di::MHCII, (clone M5/114.15.2, Biolegend, #107602), Lu175Di::CD127 (clone A7R34, Biolegend, #135002), Lu176Di::CX3CR1 (clone SA011F11, Biolegend, #149002).

Brain CyTOF: Rh103Di::Viability (Fluidigm, #201103A), In113Di::Thy1.2 (clone 30-H12, Biolegend, #105302), Pr141Di::Ly6G (clone 1A8, Fluidigm, #3141008B), Nd142Di::CD11c (clone N418, Biolegend, #117302), Nd143Di::TCRb (clone H57-597, Fluidigm, #3143010B), Nd144Di:CD24 (clone M1/69, Biolegend, #101802), Nd145Di::CD69 (clone H1.2F3, Fluidigm, #3145005B), Nd146Di::F4/80 (clone BM8, Fluidigm, #3146008B), CD88-APC (clone 20/70), Biolegend, #135808), Sm147Di::APC (clone: APC003, Biolegend, #408002), Nd148Di::CD11b (clone M1/70, Biolegend, #101202), Sm149Di::CD19 (clone 6D5, Biolegend, #115502), Nd150Di::IgD (clone 11-26c.2a, Biolegend, #405702), Eu151Di::CD25 (clone 3C7, Fluidigm, #3151007B), Sm152Di::SiglecF (clone S17007L, Biolegend, #155502), Eu153Di::CD8 (clone 53-6.7, Fluidigm, #3153012B), Sm154Di::CD169 (clone 3D6.112, Biolegend, #142402), Gd156Di::CD14 (clone Sa14-2, Fluidigm, #3156009B), Gd158Di::CD117 (clone 2B8, Biolegend, 105802), Gd160Di::CD62L (clone MEL-14, Fluidigm, #3160008B), Dy161Di::CD103 (clone 2E7, Biolegend, #121402), Dy162Di::Ly6C (clone HK1.4, Biolegend, #128002), Ho165Di::CD45 (clone 30-F11, Fluidigm, 3147003B), Tm169Di::CD206 (clone C068C2, Fluidigm, #3169021B), Er170Di::NK1.1 (clone PK136, Fluidigm, #3170002B), Yb171Di::CD38 (clone 90, Fluidigm, #3171007B), Yb172Di::CD4 (clone RM4-5, Fluidigm, #3172003B), Yb174Di::MHCII, (clone M5/114.15.2, Biolegend, #107602), Lu175Di::CD127 (clone A7R34, Biolegend, #135002), Lu176Di::CX3CR1 (clone SA011F11, Biolegend, #149002).

FAC-sorting/Flow cytometry: CD11b-PE-Cyanine7 (clone M1/70, BioLegend, #101215), Ly6C-PerCP-Cy5.5 (clone HK1.4, BioLegend, #128027), Ly6G-PE (clone 1A8, BioLegend, #127607), B220-FITC (clone RA3-6B2, BioLegend, #103205), CD90.2-APC (clone 53-2.1, BioLegend, #140312), CD45-BV510 (clone 30-F11, BioLegend, #103137), CD11b-PerCP/Cyanine5.5 (clone M1/70, BioLegend, #101227), Ly6C-APC/Cyanine7 (clone HK1.4, BioLegend, #128025), Ly6G-eFluor™450 (clone 1A8-Ly6g, Thermo Fisher Scientific #48-9668-82), CD45.1-PE-Cyanine7 (clone A20, Invitrogen, #25-0453-81), CD45.2-BV421 (clone 104, BD Bioscience, #562895), CD16/32 (clone 2.4G2, Bio X Cell, #BE0307), CD45-V500 (Fisher Scientific, #BDB560779), CD19-PE-Cy7 (Fisher Scientific, #BDB560911), CD24-PE (Fisher Scientific, #BDB560991), CD27-APC (Fisher Scientific, #BDB337169), CD38 PerCP-Cy5.5 (Fisher Scientific, #BDB551400), IgD-V450 (Fisher Scientific, #BDB561309).

Immunohistochemistry: RFP (Rockland, #600-401-379), RFP (Rockland, #200-101-379), NeuN (1:500, Abcam, #ab177487), CD31 (1:300, Biolegend, #102501), AQP4 (Thermo Fisher Scientific #PA5-85767), Cy2 (Jackson Immunoresearch, #711-225-152), Cy5 (Jackson Immunoresearch, #712-175-153), Oregon Green® 488 conjugate of NeutrAvidin® biotin-binding protein (Thermo Fisher Scientific, #A6374).

Immunoblotting: Aggrecan (Sigma-Aldrich, #AB1031), HRP conjugated β-Actin (Cell Signaling, #12262), anti-rabbit IgG HRP-linked (Cell Signaling, #7074).

Validation

All antibodies were validated for the indicated species and applications by the manufacturer.

Animals and other organisms

Policy information about studies involving animals; ARRIVE guidelines recommended for reporting animal research

Laboratory animals

The following mouse strains were used: For standard chronic social defeat stress (CSDS) experiments, 7 week-old C57BL/6J (Stock#: 000664) mice were purchased from The Jackson Laboratory. For bone marrow transplantation experiments, 4 week-old B6.SJL-Ptprca Pepcb/BoyJ (Stock#: 002014, B6 CD45.1) mice were ordered from The Jackson Laboratory. B6.129(Cg)-Ccr2tm2.1Ifc/J (STOCK#: 017586, Ccr2rfp) and B6.129X1-Mmp8tm1Otin/J (Stock#: 005514, Mmp8-/-), were bred inhouse. 4-6 month-old male retired CD-1 breeders (Charles River Laboratories, Crl:CD1[ICR]) were used as aggressors for male CSDS. For the female CSDS

	experiment, male B6N.129S6(Cg)-Esr1tm1.1(cre)And/J (Stock#: 017911, ERα-Cre) mice were purchased from The Jackson Laboratory and were crossed with CD-1 females to obtain F1 males, which were used as aggressors.
Wild animals	N/A
Field-collected samples	N/A
Ethics oversight	All procedures were performed in accordance with the National Institutes of Health Guide for Care and Use of Laboratory Animals and the Icahn School of Medicine at Mount Sinai (ISMMS) Institutional Animal Care and Use Committee.

Note that full information on the approval of the study protocol must also be provided in the manuscript.

Human research participants

Policy information about studies involving human research participants

Population characteristics	Detailed population characteristics are shown in Supplementary Tables 3 and 4. Groups did not differ in age, gender or body mass index.
Recruitment	Study participants with major depressive disorder and healthy control subjects, as assessed by the Structured Clinical Interview for the Diagnostic and Statistical Manual of Mental Disorders–Fifth Edition (SCID-5), were recruited through the Depression and Anxiety Center for Discovery and Treatment at the Icahn School of Medicine at Mount Sinai. The ISMMS review board approved the study, and written informed consent was obtained from all participants prior to any study procedure. Participants were compensated for their time and effort. Subjects provided demographic information and underwent a psychiatric evaluation using the SCID-5 conducted by trained study staff. Participants completed the Quick Inventory of Depressive Symptomatology-SR (QIDS-SR) to measure depressive symptom severity. The Perceived Stress Scale, a 10-item self-rating scale, was used to determine perceived stress levels. All participants underwent biochemistry and hematological laboratory testing, urine toxicology and pregnancy (if applicable) testing. At the time of enrollment, all participants were free of medications known to affect the immune system for at least two weeks. Participants were free of active infections or systemic illness. Subjects with concomitant unstable medical illnesses were excluded. Participants were free of current substances of abuse.
Ethics oversight	The ISMMS review board approved the study, and written informed consent was obtained from all participants prior to any study procedure.

Note that full information on the approval of the study protocol must also be provided in the manuscript.

Flow Cytometry

Plots

Confirm that:

☒ The axis labels state the marker and fluorochrome used (e.g. CD4-FITC).

☒ The axis scales are clearly visible. Include numbers along axes only for bottom left plot of group (a 'group' is an analysis of identical markers).

☒ All plots are contour plots with outliers or pseudocolor plots.

☒ A numerical value for number of cells or percentage (with statistics) is provided.

Methodology

Sample preparation	Blood: Trunk blood was collected directly into FACS buffer (DPBS (Thermo Fisher Scientific, #14190144) containing 0.5% bovine serum albumin (Sigma Aldrich, #A9647) and 2 mM EDTA (Invitrogen, #AM9260G). Samples were centrifuged and RBC lysis was performed (BD, #555899). After washing the cell pellet with ice-cold DPBS, Fc receptor blocking (rat anti-CD16/CD32, clone 2.4G2, BD Biosciences, #553141) was performed on ice for 30 min. Cells were pelleted and washed once. Cells were then stained with the antibodies listed above.<br>Brain: Mice were euthanized by injecting 10% chloral hydrate and perfused transcardially with ice-cold 0.1 M PBS (pH 7.4). Brains were rapidly dissected and put in ice-cold PBS (for brain-infiltrating monocyte RNA-sequencing experiment) or bilateral NAc tissue punches were obtained from 1 mm thick coronal slices using 1.2 mm punches (for resident myeloid cell RNA-sequencing experiment) (GE Healthcare Life Sciences, #1205X41). All the following steps were performed strictly on ice. For whole brains, tissue was cut into small pieces, for punches no shredding was needed. Tissue was then transferred to DPBS and homogenized with pestles (Sigma, #D8938-1) in ice-cold PBS (20 stokes with pestle A, 20 stokes with pestle B). The cell suspension was then passed through a 70 µm cell strainer (pre-wet with PBS) (Miltenyi Biotec, #130-095-823) into a 15 mL conical tube. Cells were pelleted (300 g for 5 min at 4 °C), resuspended in 10 mL of ice-cold 40% isotonic Percoll (Millipore Sigma, GE17-0891-01) (diluted in PBS) and centrifuged for 30 min at 500 g at 4 °C with full acceleration and braking. The myelin layer was aspirated, then the cell pellet was washed with 10 mL of ice-cold PBS by centrifuging at 300 g for 5 min at 4 °C. Cells were then resuspended in FACS buffer and Fc receptor binding was blocked (rat anti-CD16/CD32, clone 2.4G2, BD Biosciences, #553141). Cells were then stained with the antibodies listed above.
Instrument	FACSAria II (BD Biosciences)<br>LSRFortessa X-20 (BD Biosciences)

Software	FlowJo software (FlowJo LLC, Version 10.6.2)<br>FACSDiva software (BD Biosciences)
Cell population abundance	N/A
Gating strategy	Gate boundaries for positive and negative populations were defined based on single color controls and "fluorescence minus one" controls. Specific gating strategies are shown in the following Extended Data Figures:<br>- FAC-sorting of Ly6Chigh monocytes, Ly6Clow monocytes, T cells, B cells (Extended Data Figure 3c)<br>- FAC-sorting of brain-infiltrating monocytes (Extended Data Figure 5c)<br>- FAC-sorting of brain-resident myeloid cells (Extended Data Figure 6d)

☒ Tick this box to confirm that a figure exemplifying the gating strategy is provided in the Supplementary Information.
