# Supplementary material for: Circulating myeloid-derived MMP8 in stress susceptibility and depression
Source: Nature. 2024 Feb 7;626(8001):1108–15. doi: 10.1038/s41586-023-07015-2 (PMC10901735; doi:10.1038/s41586-023-07015-2)
Supplement: Supplementary file 5 — This file contains the sociodemographic variables and clinical data of the individuals shown in Fig. 1d–g. [file 41586_2023_7015_MOESM5_ESM.docx]

|  | **HC (n = 52)** | **MDD (n = 131)** | **Statistics** |
| --- | --- | --- | --- |
| **Age** | 37.37 ± 8.88 | 38.60 ± 11.76 | t(181) = 0.681,  *P* = 0.497 |
| **Gender (m/f/o)** | (33/19/0) | (68/61/2) | χ2 = 2.550,  *P* = 0.279 |
| **BMI** | 25.54 ± 4.51 | 26.25 ± 5.19 | t(180) = 0.862,  *P* = 0.390 |
| **QIDS-SR total** | 1.79 ± 2.66 | 17.84 ± 7.52 | t(181) = 14.99,  ***P*< 0.001** |

**Sociodemographic variables and clinical data.** Statistics: two-tailed Student’s t-test (for age, BMI and QIDS), Pearson's chi-squared test (for gender). Abbreviations: BMI: Body Mass Index; f: Female; HC: Healthy controls; m: Male; MDD: Major depressive disorder; o: Others; QIDS-SR: Quick Inventory of Depressive Symptomatology-Self Report.
