# Supplementary material for: Circulating myeloid-derived MMP8 in stress susceptibility and depression
Source: Nature. 2024 Feb 7;626(8001):1108–15. doi: 10.1038/s41586-023-07015-2 (PMC10901735; doi:10.1038/s41586-023-07015-2)
Supplement: Supplementary file 7 — This file contains the sociodemographic variables and clinical data of the individuals shown in Fig. 3c,d. [file 41586_2023_7015_MOESM7_ESM.docx]

|  | **HC (n = 29)** | **MDD (n = 40)** | **Statistics** |
| --- | --- | --- | --- |
| **Age** | 36.66 ± 8.57 | 34.38 ± 10.28 | t(67) = 0.974,  *P* = 0.334 |
| **Gender (m/f/o)** | (18/11/0) | (16/23/1) | χ2 = 3.693,  *P* = 0.158 |
| **BMI** | 25.23 ± 3.58 | 25.15 ± 4.80 | t(66) = 0.068,  *P* = 0.946 |
| **QIDS-SR total** | 1.45 ± 1.68 | 13.05 ± 4.37 | t(67) = -13.556,  *P* < 0.001 |

**Sociodemographic variables and clinical data.** Statistics: two-tailed Student’s t-test (for age, BMI and QIDS), Pearson's chi-squared test (for gender). Abbreviations: BMI: Body Mass Index; f: Female; HC: Healthy controls; m: Male; MDD: Major depressive disorder; o: Others; QIDS-SR: Quick Inventory of Depressive Symptomatology-Self Report.
